# Changes in mental health outcomes of middle-aged and older adults during the COVID-19 pandemic: A scoping review

**DOI:** 10.1371/journal.pone.0342140

**Published:** 2026-08-27

**Authors:** Shristi Sharma, Hiba Elhassan, Kelly K. Anderson, Marnin J. Heisel, Piotr Wilk, Joel Gagnier

**Affiliations:** 1 Department of Epidemiology and Biostatistics, Western University, London, Canada; 2 Department of Psychiatry, Western University, London, Canada; 3 Department of Epidemiology and Population Studies, Medical College, Jagiellonian University, Kraków, Poland; 4 Department of Surgery, Western University, London, Canada; Nanjing University, CHINA

## Abstract

The COVID-19 pandemic negatively impacted mental health, particularly among middle-aged and older adults, who have emerged as especially vulnerable. This scoping review synthesizes findings from 44 population-based studies retrieved from six databases, aiming to map changes in mental health outcomes during the pandemic and identify associated factors. During the global spread and response phase of the pandemic (May 2020–December 2021), a notable rise in depressive symptoms, anxiety symptoms, stress, distress, loneliness, and poor well-being was documented, highlighting a decrease in overall mental health of middle-aged and older adults. Whereas some studies from the transition period toward the endemic phase of the pandemic (2022–2023), began reporting signs of stabilization or improvement, particularly in depressive symptoms, anxiety symptoms and stress. Factors influencing these negative mental health outcomes were multidimensional (demographic, socioeconomic, health-related, behavioral, and COVID-19-related factors). Female sex, old age, being married, unemployment, lower education status, higher income, poor physical health, limited physical activity, perceived threat of COVID-19, social isolation, and lack of support were mainly associated with worsening mental health changes during the pandemic among middle-aged and older adults. However, there is little research highlighting population-level factors (COVID-19 cases, deaths, vaccination status, etc.) associated with these changes. Also, the evidence remains limited regarding mental health changes during the transition to the endemic phase, possibly due to shifts in research priorities, but it is essential to continue monitoring mental health outcomes to track long-term changes and to prepare for future public health emergencies.

## Introduction

Pandemics, environmental catastrophes, and social disasters impact the mental health of individuals [1,2]. The COVID-19 pandemic, for instance, led to disruptions in the daily lives of individuals worldwide, with governments in nearly every part of the globe implementing extensive measures to control the spread of the virus, as documented by the World Health Organization [3]. Whereas much attention was given to infection rates and mortality, the pandemic also brought about significant challenges to mental health as people faced rapid changes, uncertainty, and social isolation [4], much of which persisted into the post-COVID period [5]. Older adults were particularly at risk of worsening mental health during the pandemic due to possible functional declines impacting their ability to participate in activities of daily living, the negative impact of social isolation, and health-related fears [6,7], whereas middle-aged adults also faced worsening symptoms from pandemic-related stressors, such as social isolation, job insecurity, health worries, and an increase in caregiving responsibilities for children and parents [7]. These challenges also intensified pre-existing midlife concerns among middle-aged adults [8,9]. For instance, nearly one-third of adults aged 55 and older in the United States (US) screened positive for depressive symptoms (32%) and anxiety symptoms (29%) in the initial months of the pandemic, indicating elevated symptoms [8].

The nature of the effect of the pandemic on mental health outcomes among middle-aged and older adults changed as the pandemic progressed. The course of the pandemic can be divided into three distinct phases [9,10]; each of which introduces unique challenges to the mental health of middle-aged and older adults. During the initial outbreak and containment phase (December 2019–March 2020), most population-based data showed little evidence of widespread change in mental health indicators. This period is often used as a baseline for measuring later changes, though it is important to note that the pandemic was not widely recognized as a global public health emergency until March 2020. As a result, significant shifts in mental health outcome measures were generally only starting to be observed only during the global spread and response phase (March 2020–December 2021), with studies reporting increased depressive symptoms and a sharp rise in loneliness [11]. During the transition to the endemic phase (Jan 2022–May 2023), partial improvement was observed, where depressive symptoms scores decreased, and anxiety symptoms scores plateaued [11]. These findings highlight the dynamic nature of mental health in middle-aged and older adults during the pandemic and underscore the need for research focused on changes in mental health during the pandemic. It is crucial to understand the changes within-person in various mental health outcomes, in addition to changing prevalence levels at the population level.

This scoping review thus aims to summarize findings from original studies that reported changes in mental health outcomes among middle-aged and older adults during different phases of the pandemic, covering a broad range of mental health outcomes such as depressive symptoms, anxiety symptoms, loneliness, distress, stress, and well-being. In addition, by identifying associated factors for changes in these outcome variables, this review aims to provide a comprehensive understanding of the pandemic’s impact on mental health. The past literature reviews on the effects of the pandemic on mental health focused primarily on the prevalence rather than changes in depressive and anxiety symptoms, often overlooking other critical indicators of mental health outcomes such as loneliness, stress, and distress [12–15]. Additionally, most reviews conducted included articles published early in the pandemic, missing insight into long-term trajectories in mental health outcomes and potential recovery processes [12–14,16]. Taken together, these gaps highlight the need for a review that moves beyond prevalence estimates to examine changes in a broader range of mental health outcomes over the full course of the pandemic.

## Methods

We adopted the Arksey and O’Malley framework for conducting this scoping review [17], and the reporting was guided by the PRISMA-ScR guidelines (S1 Checklist). No review protocol was registered, and no formal assessment of methodological quality (risk of bias) of the included studies was performed [18].

### Research questions

Two key questions guided this scoping review:

1. What changes were reported among middle-aged and older adults during the COVID-19 pandemic across different mental health outcomes (depressive symptoms, anxiety symptoms, loneliness, distress, stress, and well-being)?
2. What factors are associated with adverse changes in specific mental health outcomes in this population?

### Identifying relevant studies

We developed a comprehensive search strategy using the Population–Concept–Context (PCC) framework to guide study eligibility. After consultations with the librarian and research team, relevant databases were identified: CINAHL, MEDLINE, EMBASE, PsycINFO [Subject heading databases], Scopus, and Web of Science [keyword databases]. The search was conducted on March 1, 2024, with subsequent updates on January 25, 2025, and September 8, 2025. The search was conducted using a combination of keywords, and medical subject headings obtained by entering MeSH or Emtree terms in the related database with keywords related to the COVID-19 pandemic, middle-aged, older adults, and mental health outcomes (depressive symptoms, anxiety symptoms, loneliness, stress, distress, and well-being) (S1 Appendix). Additionally, we conducted forward and backward citation searches by reviewing the reference lists of relevant articles and identifying subsequent studies that cited them, to ensure comprehensive coverage of the literature.

We included population-based studies that are quantitative, cross-sectional, or longitudinal, focusing on participants aged 45 and older (including sub populations of adults 45+). These studies reported changes in mental health outcomes such as depressive symptoms, anxiety, loneliness, distress, stress, and well-being during the COVID-19 pandemic using the same measurement scales. This criterion was applied to ensure that observed changes in mental health outcomes were attributable to changes in mental health status rather than variations in measurement resulting from the use of different scales. We excluded qualitative studies, reviews, interventional, grey literature, case studies, non-English, or those focusing on specific subgroups like dementia or COPD patients. Additionally, studies conducted solely in hospitals, clinics, universities, or home care settings were not included.

### Study selection

One reviewer (SS) first conducted a pilot screening of 10 studies to check how well the eligibility criteria were applied. After that, screening was done in two steps by two reviewers (SS and HE): (1) screening titles and abstracts, and (2) reviewing full-text articles. Both reviewers worked independently at each step. Any disagreements were resolved through discussion. Forward and backward citation searches were conducted by one reviewer (SS) to find additional relevant articles.

### Data extraction

We developed data extraction forms in MS Excel**.** The form included information on article author name, topic, year, study design and setting, timeline, change in different mental health outcomes, demographics information (age, sex, marital status, and others), socioeconomic status (education, income, employment, and others), health related factors (general health, comorbidities, and others), behavioral factors (smoking, alcohol, activities of daily living [ADL], and others), COVID-related factors, living arrangements, geography/environmental factors, and others associated with changes in mental health outcomes, measurement instruments for mental health outcomes, and main results.

The data extracted were then summarized to identify changes in specific mental health outcomes across the three phases of the COVID-19 pandemic (S1 Fig). Presenting the results by specific mental health outcomes rather than by phases of the pandemic aligns with the review’s objectives by providing a clearer understanding of how these outcomes changed over time and which factors influenced these changes among middle-aged and older adults.

## Results

**Fig 1:**
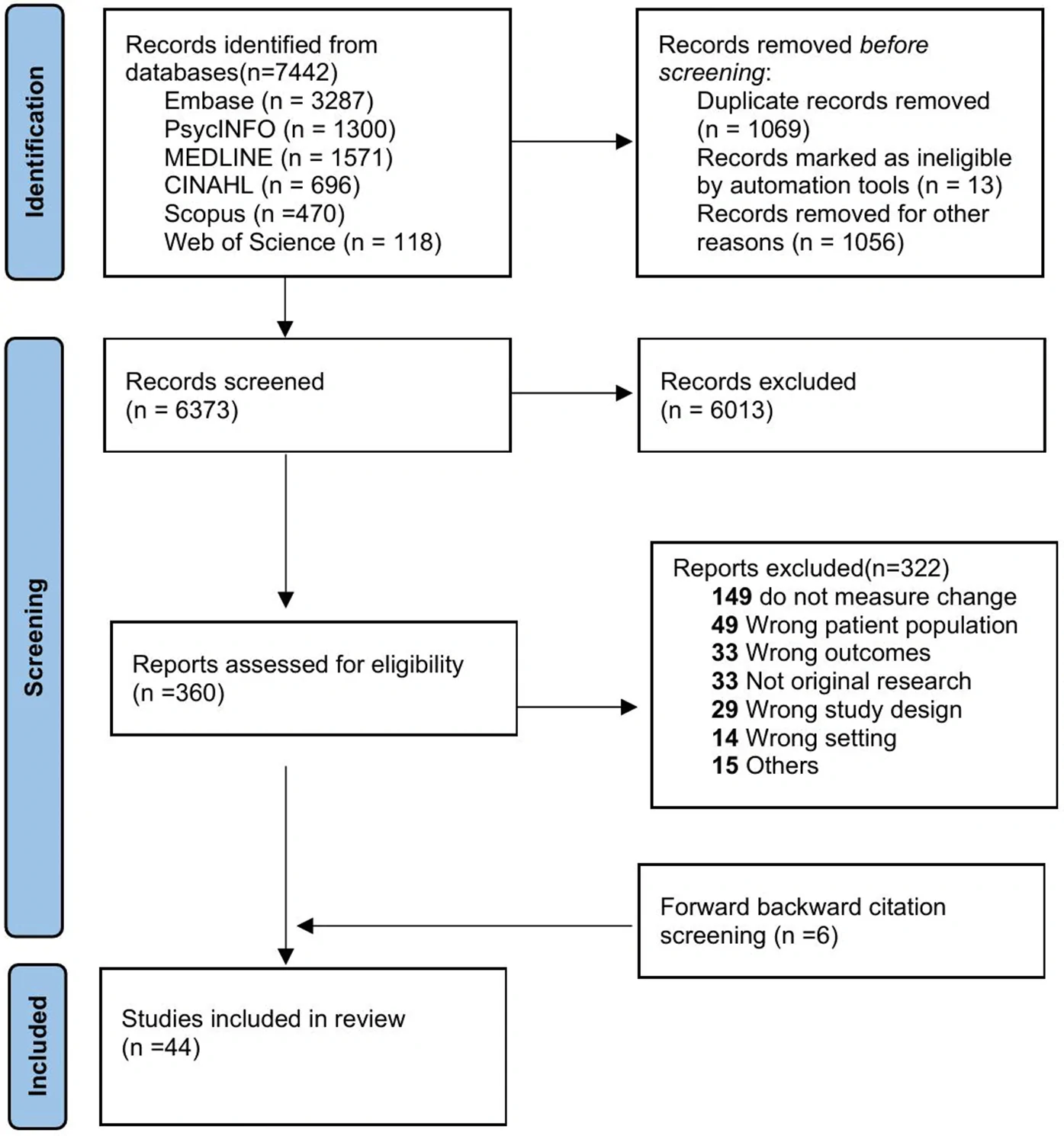
PRISMA flow diagram summarizing search process and source selection.

A total of 7,442 records were identified through initial and updated database searches. After the removal of 1069 duplicates, 6,373 records remained for title and abstract screening. Of these, 6013 were excluded based on relevance, leaving 360 full-text articles for eligibility review. Inter-rater agreement during the screening process was measured using Cohen’s kappa, with a value of 0.72, indicating substantial agreement. Following a detailed assessment, 322 articles were excluded for not meeting the inclusion criteria. An additional six studies were identified through citation tracking, resulting in 44 studies that were ultimately included in the review (Fig 1).

### Study characteristics

Table 2 summarizes characteristics of studies on changes in mental health outcomes during the COVID-19 pandemic. Geographically, most studies were conducted in Europe (45.5%) and North America (31.8%), with the US (25.0%), Canada (11.4%), the United Kingdom (11.4%), and China (9.1%) being the most frequently studied countries. Most of these studies were published in 2022 (31.8%) and 2023 (29.6%). The primary mental health outcomes studied were depressive symptoms (68.2%), loneliness (34.1%), and anxiety symptoms (31.8%), with a total of 20 unique scales used to measure health outcomes. The majority of studies (81.8%) were prospective longitudinal cohort studies, while the remaining (20.5%) were cross-sectional studies in which participants retrospectively reported their pre-pandemic mental health status to assess change. Studies reported a wide range of follow-up periods: the shortest was approximately four months, and the longest extended to nine years. Out of 44 studies, 36 used data from pre-pandemic cohorts (Table 1).

**Table 1:** Characteristics of included studies (n = 44).

Characteristics	No of Studies	Percentage of Study
**Country**		
United States	9	20.4%
Canada	5	11.4%
United Kingdom	5	11.4%
China	4	9.1%
Germany	2	4.5%
Austria	2	4.5%
Netherlands	2	4.5%
Others	12	27.4%
Multi-country studies	3	6.8%
**Continent**		
Europe	20	45.5%
North America	14	31.8%
Asia	7	15.9%
South America	2	4.5%
Oceania	1	2.3%
**Study design**		
Cross-sectional	8	18.2%
Longitudinal	36	81.8%
**Mental health outcomes**		
Depressive symptoms	30	68.2%
Loneliness	15	34.1%
Anxiety symptoms	14	31.8%
Well-being	1	2.3%
Stress	3	6.8%
Distress	2	4.6%
**Year of studies**		
2020	3	6.8%
2021	6	13.6%
2022	14	31.8%
2023	13	29.6%
2024	5	11.4%
2025	3	6.8%
**Follow-up time in months**	**Maximum**	**Minimum**
Depressive symptoms	108	6
Loneliness	60	4
Anxiety symptoms	108	6
Well-being	12	12
Stress	36	13
Distress	15	15
**Unique scale used**	**Number**	**Names**
Depressive symptoms	6	PHQ, CESD, BDI, GDS, HADS, single question
Anxiety symptoms	5	GAD, BAI. HADS, GAI, HAS, single question
Loneliness	4	UCLA, DeJong Gierveld, single question,
Well-being	1	WHO-5
Stress	3	Louisville Older Persons Event Scale, Primary Care PTSD Screener, single question
Distress	1	K10

Abbreviations: PHQ (Patient Health Questionnaire), CESD (Center for Epidemiological Studies Depression Scale), BDI (Beck Depression Inventory), GDS (Geriatric Depression Scale), HADS (Hospital Anxiety and Depression Scale), GAD (Generalized Anxiety Disorder Scale), BAI (Beck Anxiety Inventory), HAS (Hamilton Anxiety Scale), UCLA (UCLA Loneliness Scale), De Jong Gierveld (De Jong Gierveld Loneliness Scale), WHO-5 (World Health Organization – Five Health outcomes Index), K10 (Kessler Psychological Distress Scale), GAI(Geriatric Anxiety Scale).

### Changes in mental health outcomes

#### Depressive symptoms.

Twenty three studies out of 30, reported changes in depressive symptoms during the global spread and response phase, compared to pre-pandemic levels or the initial outbreak phase, with 17 out of 23 indicating an increase in depressive symptoms [7,19–34]. However, three studies reported a decrease [35–37], and three reported no significant change in depressive symptoms [38–40]. Studies measuring changes within the global spread and response phase revealed complex trends. For instance, a study by Wang and colleagues found that depressive symptoms remained stable from May to November 2020 [41]; whereas a study by Wister and colleagues reported an increase in depressive symptoms between April and December 2020 [42]. Further, a study by Mistry and colleagues also reported an increase from October 2020 to December 2021 [43]; Gerhards and colleagues reported increase from May 2020 to May 2021 [11]; and a study by Mayerl and colleagues showed an increase from May 2020 to December 2021 [44]. In contrast, Fields and colleagues reported a decrease in depressive symptoms between May 2020 and April 2021 [45]. In the transition to the endemic phase, limited studies (n = 2) indicated signs of recovery, with both and Gerhards and Pynnönen reporting decreases in depressive symptom scores [11,26]; and one study reported that depressive symptoms increased in 2022, after having decreased in 2021 compared to 2020 [46] (Table 2).

**Table 2:** Changes in mental health outcomes indicators during different phases of COVID-19.

Study (Author, Year)	Study Design	Country	Timeline	Outcomes Measured	Sample Size	Statistical Analysis for Change	Key Findings (Changes)
Amerio et al., 2023	Cross-sectional	Italy	Nov 2019, Nov 2020	Depressive symptoms (PHQ-2), Anxiety symptoms (GAD-2)	4,400	Percentage Change	Increase in depressive symptoms and Anxiety symptoms of 112% and 136%. ↑
Ang, 2022	Longitudinal	United States	2016, 2020	Depressive symptoms (CESD-8)	4,587	Paired t-test	Significant decrease in symptoms (1.22 ± 1.83 in 2016 vs 1.18 ± 1.70 in 2020). ↓
Arpino et al., 2022	Cross-sectional	Multi countries	2019 to March 2020, Jun to Aug 2020	Loneliness (single question)	35,068	Prevalent difference	The prevalence of feeling lonely only increased by 0.8%. Also, 11.6% perceived themselves as lonelier in 2020. ↑
Asada et al., 2023	Longitudinal	Canada	April 2020, December 2020	Anxiety symptoms (GAD-7)	21,130	Wilcoxon Signed Rank Test	Remain unchanged. →
Briggs et al., 2021	Longitudinal	Ireland	2016, 2018, July-Nov 2020	Depressive symptoms (CESD-8)	3,490	Multilevel regression	There was no change in CES-D scores between 2016 and 2018 → , but a large increase (β = 2.20, 95% CI 2.07, 2.33) in symptoms was observed during the pandemic in 2020 compared to 2016. ↑
Cabib et al., 2023	Longitudinal	Chile	Dec 2019 to March 2020, August 2020 to Dec 2020	Depressive symptoms (PHQ-9)	253	Prevalent difference	The prevalence of people with Depressive symptoms increased after COVID-19 (14.5% vs 23.9%). ↑
Chen et al., 2025	Longitudinal	China	2018 and 2020	Depressive symptoms (CESD-8)	7,592	Percentage	Between 2018 and 2020, 11.85% of participants aged 65+ and 12.20% of those aged 50–64 without depressive symptoms in 2018 developed depression ↑ , while 11.26% of the 65 + group and 11.85% of the 50–64 group with depressive symptoms in 2018 no longer reported symptoms in 2020 ↓ .
Compernolle et al., 2022	Longitudinal	United States	2018-2019 (3 waves), 2020 (3 waves)	Real-time loneliness (1–4) EMAs (ecological momentary assessments) level	110 (13,422 EMAs)	Multilevel regression	Increase during pandemic than pre-pandemic (β = 0.03, 95% CI 0.01, 0.04). ↑
Creese et al., 2021	Longitudinal	United Kingdom	2019, May 2020	Depressive symptoms (PHQ-9), Anxiety symptoms (GAD-7)	3,281	Case-level differences	Increase in both moderate to severe anxiety symptoms (1.8% in 2019 vs 2.7% in 2020) and depressive symptoms (4.15% in 2019 vs 5.6% in 2020). ↑
Dhakal et al., 2023	Cross-sectional	United states	June-Oct 2020	Loneliness (recall)	2,564	Bivariate analysis/ Multinomial logistic	22.16% reported feeling lonely more often. ↑
Ejiri et al., 2021	Longitudinal	Japan	June 2019, June 2020	Mental well-being (WHO-5)	618	ANCOVA	Significant decrease (16.7 ± 4.8 in 2019 vs 15.0 vs 5.2 in 2022). ↑
Eliasen et al., 2022	Longitudinal	Faroe Island	December 2017 to January 2019, 8 June to 15 July 2020	Loneliness (single question)	227	McNemar’s test	Increase in loneliness (21.8% vs. 6.8%) ↑.
Elliott et al., 2025	Longitudinal	United states	March–April 2020, September–October 2020, November 2021, and May–June 2022	Post traumatic stress (Primary Care PTSD Screener), Depression (BDI), Anxiety (BAI)	2,385	Multilevel model	PTS symptoms decreased from 2020 to 2021 and again in 2022 ↓ . Anxiety symptoms decreased in 2021 compared to 2020↓ but increased again in 2022. Depressive symptoms increased in late 2022 ↑ , then decreased in 2021 ↓ , and rose again in 2022↑
Fields et al., 2022	Longitudinal	United states	10-time bins from March 2020 to April 2021	Depressive symptoms (PHQ-9), Stress (& point Likert scale)	161	Linear Mixed Model	Depressive symptoms decreased across the course of the pandemic. ↓
Fuller & Huseth-Zosel, 2022	Cross-sectional	United states	Prior (recall), March-April 2020	UCLA (recall)	76	Paired t test	Significant increase (4.68 ± 1.81 prior vs 4.68 ± 1.81 current) loneliness. ↑
Gerhards et al., 2023	Longitudinal	Germany	May-June 2020, March-May 2021, Jan 2022	Depressive symptoms (BDI), Anxiety symptoms (BAI)	135	Multilevel model	Increase in depressive symptoms (IRR = 0.03, 95% CI 3.61, 8.14), and anxiety symptoms (IRR = 7.93, 95% CI 5.21, 12.07) from T1 to T2 ↑ , while remaining the same in T3 → .
Greenwood-Hickman et al., 2024	Longitudinal	United states	2016 to 2020, March/July 2021	Depressive symptoms (CESD)	896	Paired t-test	Depressive symptoms increase (3.7 ± 4.1 pre-pandemic vs 5.2 ± 4.9 pandemic) over time ↑ .
Gyori, 2023	Cross-sectional	Hungary	June-August 2020	Depressive symptoms (single recall question), Anxiety symptoms (single question), Loneliness (single question)	980	Percentage	Suffer from depressive symptoms (11.5%), anxiety symptoms (13.5%), and loneliness (5.6%) even more compared to before to COVID-19 (by recall). ↑
Hansen et al., 2021	Longitudinal	Norway	September–18 October 2019, January–16 February 2020	Loneliness (Single question)	10,740	Paired t-test	Remains quite stable over time. →
Holwerda et al., 2023	Longitudinal	Netherland	2018-2019, June 2020, March 2021	Depressive symptoms (CESD-10), Anxiety symptoms (HADSA), Loneliness (De Jong Grieved scale)	899	Paired t-test	Compared to pre-pandemic times, all their outcomes increased in T1 ↑ (mean change scores 1.46, 0.77, and 1.60, respectively), remaining stable in T2. →
Jeong et al., 2024	Longitudinal	Korea	2017, 2018, 2019, 2020, 2021	Depressive symptoms (CESD-11)	2,716	Growth mixture model	Steadily increased over time: 89.4% were categorized as a steadily increasing type. ↑
Kirkland et al., 2023	Longitudinal	Canada	2015-2018, 2020	Loneliness (UCLA-3)	24,114	Weighted generalized estimating equations	The prevalence of loneliness increases (50.5% during the pandemic vs 30.75% pre-pandemic) ↑
L et al., 2022	Longitudinal	Brazil	August 2019 to March 2020, May – Oct 2020 (three time points)	Loneliness (single question)	5,108	Mixed-effects logistic regression	Decrease during the pandemic compared to the pre-pandemic and initial outbreak period. 33.1% prepandemic vs 23.6%, 20.5% and 20.6% in pandemic. ↓
Li et al., 2022	Longitudinal	China	May to June 2019, August September 2020	Distress (K10)	2,749	McNemar’s test	7.28% increase in depressive symptoms. ↑
Mayerl et al., 2022	Longitudinal	Austria	May 2020, May 2021, December 2021	Depressive symptoms (BDI), Anxiety symptoms (BAI), Loneliness (UCLA-3)	370	Non-linear growth curves	Increase in Anxiety symptoms, depressive symptoms, and loneliness by 19%. ↑
McLean et al., 2025	Longitudinal	New Zealand	August-November 2018, June-September 2020	Depressive symptoms (CESD-10), Anxiety Symptoms (GAI-SF)	3,171	Paired-t test	Depressive and anxiety symptoms did not change significantly between the two time points → .
Mistry et al., 2023	Repeated cross-sectional	Bangladesh	Oct 2020, Dec 2021	Depressive symptoms (GDS-15)	2,077	Non-linear growth curve	Significant increase (OR= 1.40, 95% CI 1.11, 1.75). ↑
Musbat et al., 2024	Longitudinal	Multi countries	October 2019- March 2020, June 2020, June 2021	Depressive symptoms (single question)	31,526	McNemar’s test	Decrease in depressive symptoms (39.1 in 2019 vs 24.8% in 2020 vs 29.5% in 2021). ↓
Pynnönen et al., 2024	Longitudinal	Finland	2017-2018, 2021, 2022	Depressive symptoms (CESD-10)	608	Paired t-test	Increase from 2018(4.8 ± 4.1) to 2021 (7.8 ± 4.6) ↑ and decrease in 2022(6.4 ± 4.2). ↑
Raina et al., 2021	Longitudinal	Canada	2015-2018, April 2020, December 2020	Depressive symptoms (CESD-10)	28,559	Weighted generalized estimating equations	Increases significantly (OR=1.84, 95% CI 1.77, 1.91) in April, and (OR= 1.99, 95% CI 1.91, 2.07) in December. ↑
Richardson et al., 2023	Longitudinal	United Kingdom	2018- 2019, June-October 2020	Loneliness (Single item)	379	McNemar’s test	Significantly increased (17.2% vs 25.1%). ↑
Robb et al., 2020	Cross-sectional	United Kingdom	April-July 2020	Depressive symptoms (HDS), Anxiety symptoms (HAS)	7,127	Percentage	12.8% and 12.3% of participants reported feeling worse on the Depressive symptoms and Anxiety symptoms components. Fewer participants reported feeling improved (1.5% for Depressive symptoms and 4.9% for Anxiety symptoms). ↑
Segerstrom et al., 2023	Longitudinal	United States	January 5, 2018–January 22, 2020, January 23, 2020–October 31, 2021	Depressive symptoms (GDS), Stress (Louisville Older Persons Event Scale)	111	Linear Mixed-Effects Models	73% increase in depressive symptoms, 57% increase in stress. ↑
Stolz et al., 2021	Cross-sectional	Austria	2013, 2015, 2017, 2020	Loneliness (UCLA-3)	557	Population-level loneliness change	Loneliness median scores in 2020 were 1 point higher compared with previous years. ↑
van den Besselaar et al., 2021	Longitudinal	Netherland	2011-2013, 2015-2016, 2018-2019, 2020	Depressive symptoms (CESD-10), Anxiety symptoms (HADS-A)	1,068	Linear mixed regression	The CES-D-10 (β = 1.37, 95% CI 1.12, 1.62) and HADS-A (β = 0.74, 95% CI 0.56, 0.94) scores showed a gradual increase over time ↑ .
Wang et al., 2023	Longitudinal	China	June 2019, August to September 2020	Distress (K10)	2,749	Multilevel	Increase (β = 1.59, SE = 0.12). ↑
J. Wang et al., 2022	Longitudinal	United Kingdom	May to Nov 2020 monthly	Depressive symptoms (PHQ-9), Anxiety symptoms (GAD-7)	3,462	Interrupted time series analysis	PHQ-9 increased in June–September (3.76 ± 3.27 vs. 3.54 ± 3.32 pre-June) but returned to baseline after September (3.47 ± 3.30), while GAD-7 rose in June–September (1.76 ± 2.70 vs. 1.62 ± 2.82) and remained higher after September (1.63 ± 2.86). ↑
Wester et al., 2022	Longitudinal	Multi countries	October 2019- March 2020, June August 2020	Depressive symptoms (single question), Loneliness (single question)	36,478	Multilevel	Decrease in depressive symptoms by 14.4% ↓ , increase in loneliness by 1.2%. ↑
Wettstein et al., 2022	Longitudinal	Germany	2014, 2017, 2020	Depressive symptoms (CESD-10)	10,793	Change score models	Between 2014 and 2017, there was no significant change in the measured outcome → . However, between 2017 and 2020, the outcome increased significantly. ↑
Wister, Li, Levasseur, et al., 2023	Longitudinal	Canada	April 2020, Dec 2020	Depressive symptoms (CESD-10), Anxiety symptoms (GAD-7)	18,099	Paired t-test	Significant increase in depressive symptoms (5.79 ± 4.96 vs 5.92 ± 5.05) ↑ and decrease in anxiety symptoms (2.30 ± 3.40 vs 2.21 vs 3.20). ↓
Wister, Li, Best, et al., 2023	Longitudinal	Canada	2015-2018, Dec 2020	Depressive symptoms (CESD-10)	12,469	Multilevel	Significantly increase (4.47 ± 3.97 vs 5.34 ± 4.55) ↑
Xu et al., 2024	Longitudinal	United states	2018- 2019, 2020-2021	Depressive symptoms (CESD-10)	13,460	Multilevel	It did not change with time. →
Zaninotto et al., 2022	Longitudinal	United Kingdom	2018-2019, July 2020, December 2020	Depressive symptoms (CESD-8), Anxiety symptoms (GAD-7), Loneliness (UCLA-3)	5,136	fixed-effects regression models	Increased significantly across all time points for depressive symptoms (12.5% vs 22.6% vs 28.5%), anxiety symptoms (9.4% vs 10.9%), and mean loneliness (5.50 vs 5.65 vs 5.75). ↑
Zhu & Chou, 2024	Longitudinal	Hong Kong China	May -October 2019, May- July 2021	Depressive symptoms (GDS-15)	838	Linear latent growth model	The level of Depressive symptoms during the pandemic did not show a significant difference compared to the period before the pandemic. →

Note: ↑ increase, → no change, decrease ↓ ; Repeated cross-sectional: does not follow the same individuals over time, change in population level prevalence.

#### Anxiety symptoms.

Overall findings also revealed an increase in anxiety symptoms during the pandemic. Whereas no studies exclusively measured changes in anxiety symptoms between the pre-pandemic and the initial outbreak phase, 8 out of 9 that measured changes in anxiety symptoms during the global spread and response phase reported heightened anxiety symptoms compared to pre-pandemic levels [19,22–24,28,30,31,33], and one reported no significant changes [40]. Four studies reported changes within the global spread and response phase. For instance, a study by Wang and colleagues indicated that anxiety symptoms increased after September 2020, after remaining stable from May 2020 to August 2020 [41]; and Mayerl and colleagues reported an increase from May 2020 to Dec 2021 [44]. In contrast, one study reported relatively stable levels of anxiety symptoms from April to December 2020 [47]; and one showed a decrease from May to December 2020 [33]. Gerhards and colleagues reported an increase in anxiety symptoms from May 2020 to May 2021, which, by the endemic phase, had plateaued [11]. However, a study by Elliott and colleagues reported an increase in anxiety symptoms in 2022 as compared to 2021 [46] (Table 2).

#### Loneliness.

Loneliness emerged as a critical mental health outcome during the pandemic. Twelve studies out of 15 studies that reported changes in loneliness during the global spread and response phase compared to pre-pandemic levels showed significant increases [23,24,30,37,48–55]; two reported decreases [37,56]; and one reported no change [57] in levels of loneliness (Table 2). Further, Mayerl and colleagues found that loneliness worsened twice from May 2020 to December 2020 [44] (Table 2).

#### Other outcomes.

Ejiri and colleagues assessed psychological well-being during the pandemic and reported a significant decrease in well-being during the global spread and response phase [58] compared to pre-pandemic levels**.** Two studies measured change in psychological distress during the global spread and response phase, both reporting increases in distress symptoms compared to pre-pandemic levels [59,60]. Finally, one study reported a decrease in stress over the pandemic [45], and one reported an increase in overall stress levels [27]. In transition to endemic period, Elliott and colleagues reported decrease in post-traumatic stress in 2022 as compared to 2020 and 2021 [46] (Table 2).

### Factors associated with changes in mental health outcomes

#### Depressive symptoms.

**Fig 2:**
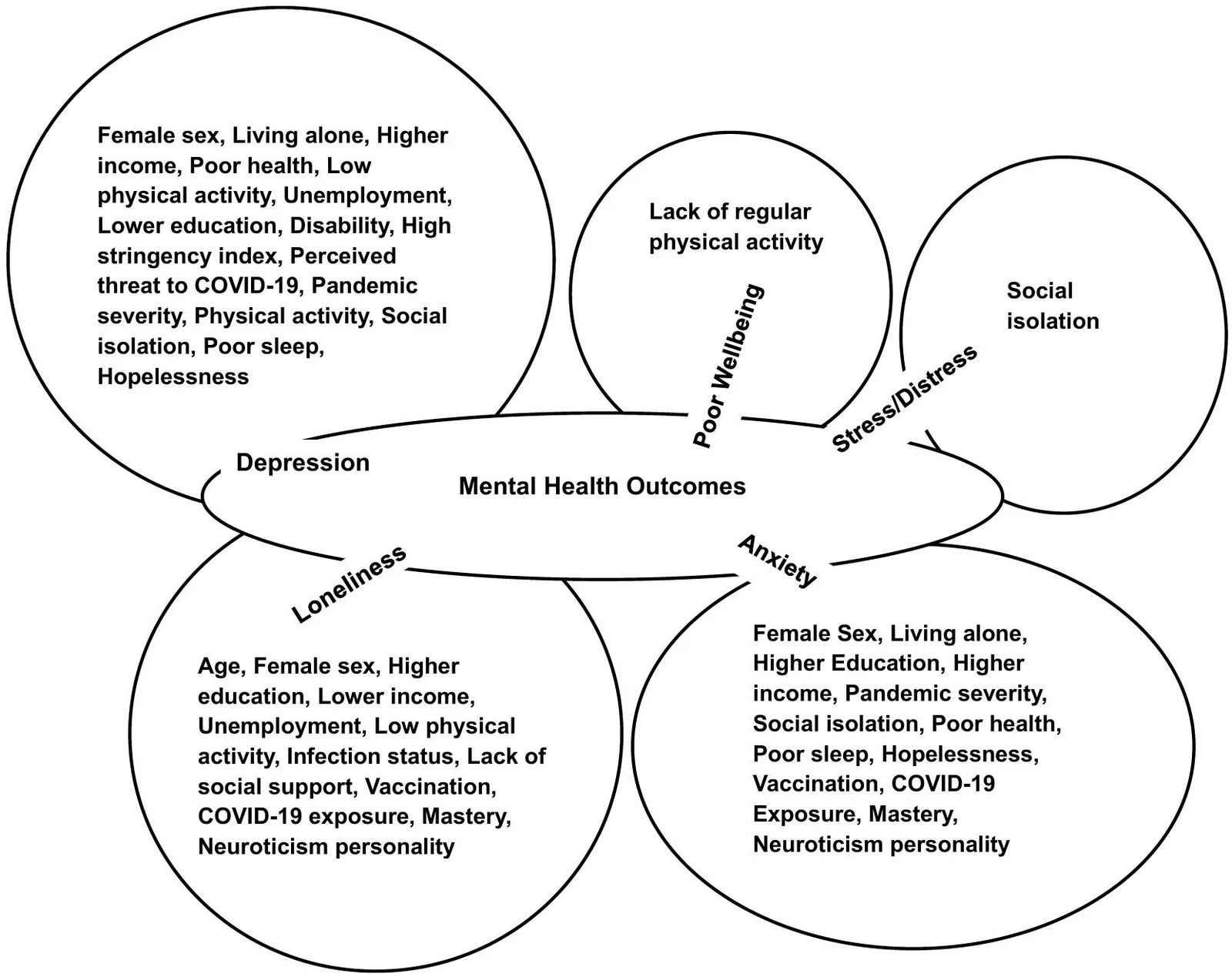
Factors positively associated with changes in mental health outcomes. Note: Factors included are those reported as positively significant by most studies; however, some evidence suggests an opposite association or no association for certain factors.

Several factors were significantly associated with the level of change in depressive symptoms during the COVID-19 pandemic. Five out of 10 studies that measure the association of age and changes in depressive symptoms found that within middle-aged and older adult populations, greater age was associated with higher depressive symptoms [20,23,27,30,37]. Female sex was associated with increased depressive symptoms over time in eight out of 13 studies examining sex [7,19,22,23,29–31,34]. Regarding socio-economic factors, four out of eight studies assessing education suggested a lower educational status [19,29,37,42]; two out of four studies assessing employment status suggested being unemployed [37,42], and three out of eight assessing income suggested a higher income [19,33,42] were associated with an increase in depressive symptoms. Specific COVID-19-related factors, such as perceived threat and pandemic severity [7,23,29,59], and a high stringency index (a measure of the strictness of government COVID-19 policies such as lockdowns, school closures, and travel restrictions) was associated with an increase in depressive symptoms [37]. COVID-19 infection status and vaccination status were, however, not associated with a change in depressive symptoms [24,37]. However, self-reported poor health was also associated with depressive symptoms in three studies [34,37,42]. Limited physical activity was associated with increased depressive symptoms in five out of seven studies assessing physical activity [22,24,34,37,42]. Social isolation/less social contact [7,22,23,31], poor sleep [31], hopelessness [19], and neurotic personality traits [24] were found to be associated with an increase in depressive symptoms (Fig 2 and S1 Table).

#### Anxiety symptoms.

Several socio-demographic factors were associated with increased anxiety symptoms. Among middle-aged and older adults, greater age was associated with higher anxiety symptoms in two out of eight studies that looked at age as an associated factor [19,23] and female sex in five out of eight studies examining sex [19,22,23,30,31]. Regarding socio-economic factors: lower education status in two out of four studies assessing education status [23,33]; being unemployed in one out of three studies assessing employment status [23]; and having high income in two out of three studies assessing income were positively associated with anxiety symptoms [19,33]. For COVID-19-related factors, one study [23] identified pandemic severity as an associated factor for anxiety symptoms. Finally, comorbidities [19,22,23] and decreased physical activity [19,22] were reported as associated factors for change in anxiety symptoms. Social isolation/less social contact [22,24,31], poor sleep [31], hopelessness [19], and neurotic personality traits [24] were found to be associated with a change in anxiety symptoms (Fig 2 and S2 Table).

#### Loneliness.

During the COVID-19 pandemic, the change in loneliness was influenced by a complex interplay of factors. Three out of eight studies examining age reported that a change in loneliness increases with age among middle-aged and older adults [23,24,55]. Six out of nine studies assessing sex reported a higher increase in loneliness in women [23,30,37,50,53,55]. One study reported being white as a associated protective factor against loneliness [53]. Residency in a rural area was negatively associated with the increase in loneliness in two out of four studies assessing rurality [50,51]. Regarding socio-economic factors, higher education was associated with increased loneliness in two out of five studies assessing education status [50,51]; and unemployment was linked to increased loneliness in three out of four studies assessing employment status [23,51,55]. For COVID-19-related factors, two studies found infection status to be associated with increased loneliness [37,49]. For health-related factors, comorbidities [23,50,55] and self-reported poor health [49] were associated with loneliness. Limited physical activity was associated with increased loneliness in two studies [24,55]. Living alone was consistently associated with increased loneliness in nine studies [23,37,48–50,53,55,56], whereas social isolation and reduced social contact were strongly associated with increased loneliness in five out of six studies examining social isolation [23,24,49,50,56]. Finally, other factors such as depression [50,53,56], mastery [24], and neuroticism [24] were also found to be associated with increased loneliness in one study (Fig 2 and S3 Table).

#### Other outcomes.

One study that looked at psychological well-being highlighted that regular physical activity, particularly walking and home training, significantly contributed to positive changes in psychological well-being. There was one study that reported factors related to differences in distress levels during the pandemic [60]. The study reported non-significant results for demographic, chronic condition, and behavioral factors. Social isolation, frailty, multimorbidity, and lower community-level social support were, however, associated with higher distress levels [60] (Fig 2).

## Discussion

This scoping review summarizes the findings from 44 studies that were published between December 2020 and September 2025; and measured changes in various mental health outcomes, including depressive symptoms, anxiety symptoms, stress, distress, loneliness, and well-being among middle-aged and older adults across different phases of the pandemic.

Overall, the COVID-19 pandemic had a significant negative impact on mental health outcomes. Most studies reported an increase in depressive symptoms, anxiety symptoms, loneliness, stress, and distress, and a decrease in well-being during the global spread and response phase of the pandemic for middle-aged and older adults. Depressive symptoms emerged as one of the most affected outcomes, followed by anxiety symptoms and loneliness. This aligns with broader narratives of pandemic disruptions [61]. By the transition to the endemic phase, although one study showed an increase in depressive and anxiety symptoms in 2022 [46], other evidence suggests improvement in overall mental health outcomes, implied by declining prevalence of depressive symptoms [11,26], decline in stress [46], and stabilized anxiety symptoms [11]. This contrasts with the narrative suggesting that the impact of COVID-19 on mental health outcomes is likely to persist even after the pandemic has officially ended [62]*.* However, limitations exist due to the paucity of research at the end of the pandemic, potentially reflecting a shift in research priorities toward other health problems. More recent assessments might still be unpublished or in progress. It is also important to note that observed changes in mental health over time could also be influenced by mortality among high-risk individuals. However, direct evidence on this issue remains extremely limited.

The substantial heterogeneity in results may be attributable to differences in countries and study designs. Across countries, increases in mental health problems were reported in many settings, including Canada, the United Kingdom, Germany, Japan, Korea, the Faroe Islands, Bangladesh, the Netherlands, Austria, Finland, Chile, and Italy. For example, the majority of Canadian, European, and Asian (excluding China) studies reported worsening mental health outcomes [19–21,23,24,26,28,33,42,44,48,50]. In contrast, studies from the United States and China showed more heterogeneous findings, with some reporting increases [32,51,53,54,59,60], and others reporting decreases or no significant changes [34,36,38,39,45,46]. Outside this region, the study from Brazil reported a decrease in loneliness during the pandemic period [56], and New Zealand showed no significant change [40]. Differences in study design may also explain variation in findings. Most longitudinal studies reported increases in mental health symptoms [20,21,24,26,28,32,33,42,44,50,54]; however, some studies observed stable or decreased symptoms [34,36,38–40,45,46,56]. In contrast, all included cross-sectional studies showed increases in mental health symptoms during the pandemic; however, interpretation is generally limited because these studies often rely on comparisons with recalled pre-pandemic status [19,23,31,43,48,51,53,55]. Overall, the direction and magnitude of changes were not uniform across countries or study designs. The variation suggests that the impact of the pandemic on mental health was influenced not only by the occurrence of COVID-19 itself but also by contextual factors like countries and study design.

This also underscores the importance of considering individual and population-level factors when interpreting changes in various mental health outcomes. Key individual-level factors associated with increases in different mental health outcomes included female sex, older age, being married, lower education, unemployment, higher income, poor health, higher perceived threat of COVID-19, low physical activity, and social isolation/lack of social support. There were similarities and differences among these common factors compared with other reviews that examined prevalence but not change over time in mental health [13–15]. Similarities include identification of female sex, social isolation, poor health/comorbidities, and unemployment as associated factors. However, differences emerged, as most studies included in our review identified higher income as an associated factor for changes in mental well-being. In contrast, in two other reviews, lower income was identified as an associated factor for the prevalence of poor mental health outcomes [13,15]. This discrepancy may reflect differences in study focus, as our review emphasizes changes over time, where individuals with higher income may have experienced greater relative declines during the pandemic, compared to the other reviews that emphasize prevalence, where lower-income groups consistently face higher absolute risk. Further, the studies included in our reviews did not support an association between the COVID-19 infection status and changes in depressive symptoms and anxiety symptoms, despite this factor being associated with various mental health outcomes in prevalence-related studies [63–65]. Thus, infection status might have impacted acute mental health, but in the longer term, the mental health of middle-aged and older adults appears to be more influenced by broader pandemic-related factors such as pandemic severity, stressors of COVID-19, and government response to COVID-19 rather than the infection status itself. However, there were limited studies examining the effects of pandemic-related factors on changes in mental health outcomes.

Further, available evidence has measured mental health changes only up to two years into the pandemic, which may be insufficient to measure the long-term mental health impact of the pandemic. Evidence from other epidemics, such as SARS-CoV-1, shows that depressive symptoms, anxiety symptoms, and PTSD can persist for many years, with studies reporting effects up to 12 years [66–68]. Similarly, in other public health crises, such as Hurricane Katrina in the US, a study showed that survivors experience persistent distress and stress symptoms up to 12 years later [69]. These examples show that the mental health effects of public health crises like pandemics or disasters often last long after the event is over, highlighting the importance of longer and more sophisticated studies to better understand and support people at risk of worsening mental health outcomes related to COVID-19.

### Strengths and limitations

The significant contribution of this scoping review is that it summarizes the available evidence on changes in mental health outcomes of middle-aged and older adults. To our knowledge, this is the first review that exclusively focused on changes in mental health outcomes of middle-aged and older adults rather than just the prevalence of these outcomes during the pandemic. Focusing on changes in mental health outcomes offers a clearer understanding of the actual impact of the pandemic on mental health. Another strength of this study is that we summarized the changes in different outcomes across all three phases of the pandemic, considering the dynamic nature of the pandemic. This review also employed a comprehensive search strategy across multiple databases, ensuring broad coverage of relevant literature. Additionally, careful and systematic data extraction was conducted to maintain accuracy and consistency. The summary of findings by specific domains of mental health further enhances the clarity and utility of this study.

Findings from this review should be interpreted considering the following limitations. Firstly, by only including English studies, the risk of language bias may limit the review’s scope and applicability. Secondly, this review included all literature that measured changes in mental health outcomes; thus, some studies may have been of low quality, potentially introducing bias into our findings. Thirdly, although we used depressive symptoms, anxiety symptoms, loneliness, stress, distress, and well-being scales as distinct outcome indicators for mental health, these measures may not fully capture the multifaceted nature of mental health. Another significant limitation of this scoping review is the inclusion of studies employing varied diagnostic tools and effect estimates, which complicates the synthesis of findings and may obscure the overall interpretability and comparability of the results across studies. Studies included in this review exhibit significant variability in both pre-pandemic measurement periods and follow-up periods, affecting cross-study comparability. For instance, a study by Besselaar and colleagues used data from 2011−2019 as a pre-pandemic baseline [28], whereas Amerio and colleagues relied on 2019 data for baseline measures [19], which may affect the comparability across studies. Notably, the grouping of middle-aged and older adults in several studies limits our ability to assess the distinct impact of the pandemic on each age group separately. Finally, although we aimed to examine a broader spectrum of mental health outcomes, our findings predominantly focused on depressive symptoms, anxiety symptoms, and loneliness. This limitation reflects the current state of research in the field, as these three outcomes have been the primary focus of most studies assessing changes in mental health during the COVID-19 pandemic. Future research should expand to include a broader range of mental health outcomes to provide a more comprehensive understanding of the impact of the pandemic.

In conclusion, the COVID-19 pandemic has significantly impacted the mental health of middle-aged and older adults, with notable increases in depressive symptoms, anxiety symptoms, and loneliness. Although some studies show mixed results for depressive symptoms and loneliness, most studies indicate a decline in mental health during pandemics. Although there is limited research conducted on mental health outcomes like stress, distress, and well-being, results from later stages of the pandemic also remain sparse. Additionally, the available literature fails to adequately examine population-level factors, such as pandemic cases, COVID-19 deaths, and vaccination status, which are crucial given the observed population-level impact. These research gaps represent a significant limitation in our understanding of the broader contextual mental health trends and factors associated with these changes during the COVID-19 pandemic among middle-aged and older adults, underscoring the need for continued investigation to guide policy development and form targeted strategies, such as age-specific interventions focusing on enhancing mental health, for better understanding, preparedness and managing of adverse mental health outcome among middle age and older adults during any future public health emergencies.

## Supporting information

S1 Fig Phases of COVID-19 and key events. (TIF)

S1 Appendix Search strategy (Medline). (DOCX)

S1 Checklist Preferred Reporting Items for Systematic reviews and Meta-Analyses extension for Scoping Reviews (PRISMA-ScR) Checklist. (DOCX)

S1 Table Factors associated with change in depressive symptoms (n = 30 studies). (DOCX)

S2 Table Factors associated with change in Anxiety Symptoms (n = 14 studies). (DOCX)

S3 Table Factors associated with change in Loneliness (n = 15 studies). (DOCX)
