# Supplementary material for: Changes in mental health outcomes of middle-aged and older adults during the COVID-19 pandemic: A scoping review
Source: PLoS One. 2026 Aug 27;21(8):e0342140. doi: 10.1371/journal.pone.0342140 (PMC13521375; doi:10.1371/journal.pone.0342140)
Supplement: Supplementary file 1 [file pone.0342140.s002.docx]

**S1 Appendix: Search strategy (Medline)**

| 1. | COVID-19.mp. or exp COVID-19/ |  |  |  |  |
| --- | --- | --- | --- | --- | --- |
| 2. | Coronavirus.mp. or exp Coronavirus/ |  |  |  |  |
| 3. | SARS-COV-2.mp. or exp COVID-19/ |  |  |  |  |
| 4. | exp Pandemics/ or Novel corona virus.mp. |  |  |  |  |
| 5. | mental health.mp. or exp Mental Health/ |  |  |  |  |
| 6. | exp Well Being/ or exp Distress/ or exp Mental Disorders/ or mental wellbeing.mp. |  |  |  |  |
| 7. | exp Anxiety Disorders/ or exp Anxiety/ or anxiety.mp. |  |  |  |  |
| 8. | exp "Depression (Emotion)"/ or exp Late Life Depression/ or exp Major Depression/ or exp Recurrent Depression/ or Depression.mp. |  |  |  |  |
| 9. | exp Stress/ or exp Psychological Stress/ |  |  |  |  |
| 10. | psychological problems.mp. |  |  |  |  |
| 11. | mental disorders.mp. or exp Mental Disorders/ |  |  |  |  |
| 12. | loneliness.mp. or exp Loneliness/ |  |  |  |  |
| 13. | PTSD.mp. or exp Posttraumatic Stress Disorder/ |  |  |  |  |
| 14. | general psychiatric distress.mp. |  |  |  |  |
| 15. | Old age.mp. or exp Older Adulthood/ |  |  |  |  |
| 16. | exp Older Adulthood/ or old* adult.mp. |  |  |  |  |
| 17. | elder*.mp. |  |  |  |  |
| 18. | middle age.mp. |  |  |  |  |
| 19. | middle-age adult.mp. |  |  |  |  |
| 20. | old people.mp. |  |  |  |  |
| 21. | Age>45.mp. |  |  |  |  |
| 22. | 1 or 2 or 3 or 4 |  |  |  |  |
| 23. | 15 or 16 or 17 or 18 or 19 or 20 or 21 |  |  |  |  |
| 24. | 5 or 6 or 7 or 8 or 9 or 10 or 11 or 12 or 13 or 14 |  |  |  |  |
| 25. | 22 and 23 and 24 |  |  |  |  |
| 26. | limit 25 to (full text and human and English language) |  |  |  |  |
