# Supplementary material for: Changes in mental health outcomes of middle-aged and older adults during the COVID-19 pandemic: A scoping review
Source: PLoS One. 2026 Aug 27;21(8):e0342140. doi: 10.1371/journal.pone.0342140 (PMC13521375; doi:10.1371/journal.pone.0342140)
Supplement: Supplementary file 3 [file pone.0342140.s004.docx]

S1 Table. Factors associated with change in depressive symptoms (n= 30 studies)

| **Factors** | **Positive- Increase in symptoms** | **Negative- Decrease in symptoms** | **Not Significant** |
| --- | --- | --- | --- |
| **Socio-demographics** |  |  |  |
| Age | (Briggs et al., 2021; Gyori, 2023; Segerstrom et al., 2023; Wester et al., 2022; Zaninotto et al., 2022) | (Raina et al., 2021; Robb et al., 2020; Wettstein et al., 2022; Wister, Li, Levasseur, et al., 2023) | (Wister et l., 2023) |
| Sex: Female | (Amerio et al., 2023; Chen et al., 2025; Creese et al., 2021; Gyori, 2023; Raina et al., 2021; Robb et al., 2020; Wettstein et al., 2022; Zaninotto et al., 2022) |  | (Briggs et al., 2021; Holwerda et al., 2023; Segerstrom et al., 2023; Wester et al., 2022; Wister, Li, Levasseur, et al., 2023) |
| Ethnicity |  |  | (Asada et al., 2023; Wister, Li, Best, et al., 2023; Wister, Li, Levasseur, et al., 2023) |
| Marital Status:Not married | (Robb et al., 2020) | (Wister, Li, Levasseur, et al., 2023) | (Creese et al., 2021; Holwerda et al., 2023; Robb et al., 2020; Wister, Li, Best, et al., 2023; Zaninotto et al., 2022) |
| Rural/ Village |  | (Gyori, 2023) | (Amerio et al., 2023; Briggs et al., 2021; Wettstein et al., 2022; Wister, Li, Levasseur, et al., 2023) |
| **Socio-economic** |  |  |  |
| Education: Higher education | (Raina et al., 2021) | (Amerio et al., 2023; Wester et al., 2022; Wettstein et al., 2022; Wister, Li, Levasseur, et al., 2023) | (Creese et al., 2021; Holwerda et al., 2023; Segerstrom et al., 2023) |
| Employment: Unemployed | (Wester et al., 2022; Wister, Li, Levasseur, et al., 2023) |  | (Creese et al., 2021; Wister, Li, Levasseur, et al., 2023) |
| Income: Less | (Chen et al., 2025; Creese et al., 2021; Raina et al., 2021) | (Amerio et al., 2023; Wister, Li, Best, et al., 2023; Wister, Li, Levasseur, et al., 2023) | (Holwerda et al., 2023; Zaninotto et al., 2022) |
| **Health** |  |  |  |
| Comorbidities | (Gyori, 2023; Raina et al., 2021) |  | (van den Besselaar et al., 2021; Wister, Li, Best, et al., 2023; Wister, Li, Levasseur, et al., 2023) |
| Self-rated health; poor | (Chen et al., 2025; Wettstein et al., 2022; Wister, Li, Levasseur, et al., 2023) |  |  |
| Disability | (Xu et al., 2024) |  | (Amerio et al., 2023) |
| **COVID-19 related factors** |  |  |  |
| Infection status |  |  | (Holwerda et al., 2023; Wester et al., 2022) |
| Stringency index: Low stringency |  | (Wester et al., 2022) |  |
| Perceived threat/stressors of COVID | (Raina et al., 2021; Wettstein et al., 2022) |  |  |
| Severity/Pandemic involvement | (Gyori, 2023; J. Wang et al., 2022) |  | (Segerstrom et al., 2023) |
| Vaccination |  |  | (Holwerda et al., 2023) |
| **Behavioral factors** |  |  |  |
| Alcohol: No drinker |  | (Raina et al., 2021; Robb et al., 2020) |  |
| Smoking: No smoker |  | (Raina et al., 2021) | (Robb et al., 2020) |
| Physical Activity(ADL/IADL/Frality): Limited activity | (Chen et al., 2025; Creese et al., 2021; Holwerda et al., 2023; Wester et al., 2022; Wister, Li, Levasseur, et al., 2023) | (Pynnönen et al., 2024) | (van den Besselaar et al., 2021) |
| **Living Situation-Loving Alone** | (Amerio et al., 2023; Briggs et al., 2021; Robb et al., 2020) |  | (van den et al., 2021; Wister, Li, Levasseur, et al., 2023) |
| **Others** |  |  |  |
| Social isolation/ less social contact/Loneliness/less internet use | (Creese et al., 2021; Gyori, 2023; Raina et al., 2021; Robb et al., 2020) |  | (Holwerda et al., 2023) |
| Caregiver |  | (Wister, Li, Levasseur, et al., 2023) | (Holwerda et al., 2023) |
| Coping strategy/Praying |  | (Pynnönen et al., 2024) | (Holwerda et al., 2023) |
| Poor sleep | (Robb et al., 2020) |  |  |
| Mastery |  | (Holwerda et al., 2023) |  |
| Hopelessness | (Amerio et al., 2023) |  |  |
