# Supplementary material for: Changes in mental health outcomes of middle-aged and older adults during the COVID-19 pandemic: A scoping review
Source: PLoS One. 2026 Aug 27;21(8):e0342140. doi: 10.1371/journal.pone.0342140 (PMC13521375; doi:10.1371/journal.pone.0342140)
Supplement: Supplementary file 4 [file pone.0342140.s005.docx]

S2 Table. Factors associated with change in Anxiety Symptoms (n=14 studies)

| Factors | Positive- Increase in symptoms | Negative: Decrease in symptoms | Not Significant |
| --- | --- | --- | --- |
| **Socio-demographics** |  |  |  |
| Age | (Amerio et al., 2023; Gyori, 2023) | (Fields et al., 2022; Robb et al., 2020) | (Creese et al., 2021; Holwerda et al., 2023; Wister, Li, Best, et al., 2023; Zaninotto et al., 2022) |
| Sex: Female | (Amerio et al., 2023; Creese et al., 2021; Gyori, 2023; Robb et al., 2020; Zaninotto et al., 2022) |  | (Holwerda et al., 2023; Wister, Li, Best, et al., 2023; Zaninotto et al., 2022) |
| Ethnicity(White) |  |  | (Asada et al., 2023; Wister, Li, Best, et al., 2023; Zaninotto et al., 2022) |
| Marital Status:Not married | (Robb et al., 2020) |  | (Creese et al., 2021; Holwerda et al., 2023; Zaninotto et al., 2022) |
| Rural/ Village |  | (Gyori, 2023) | (Amerio et al., 2023; Wister, Li, Best, et al., 2023; Zaninotto et al., 2022) |
| **Socio-economic** |  |  |  |
| Education: Higher education |  | (Gyori, 2023; Wister, Li, Best, et al., 2023) | (Creese et al., 2021; Wister, Li, Best, et al., 2023) |
| Employment: Unemployed | (Gyori, 2023) |  | (Amerio et al., 2023; Creese et al., 2021; Holwerda et al., 2023) |
| Income: Less |  | (Amerio et al., 2023; Wister, Li, Best, et al., 2023) | (Asada et al., 2023; Creese et al., 2021; Holwerda et al., 2023; Zaninotto et al., 2022) |
| **Health** |  |  |  |
| Comorbidities | (Amerio et al., 2023; Creese et al., 2021; Gyori, 2023) | (Wister, Li, Best, et al., 2023) | (van den Besselaar et al., 2021) |
| Self-rated health; poor |  |  |  |
| Disability |  |  |  |
| Prepandemic mental health |  | (Holwerda et al., 2023) | (Asada et al., 2023; van den Besselaar et al., 2021) |
| **COVID-19 related factors** |  |  |  |
| Severity/Pandemic involvement | (Gyori, 2023) |  |  |
| Vaccination |  |  |  |
| **Behavioral factors** |  |  | (Holwerda et al., 2023) |
| Alcohol: No drinker |  |  | (Robb et al., 2020) |
| Smoking: No smoker |  |  | (Robb et al., 2020) |
| Physical Activity(ADL/IADL/Frality): Limited activity | (Amerio et al., 2023; Creese et al., 2021) |  | (Holwerda et al., 2023; van den Besselaar et al., 2021) |
| **Living Situation-Living Alone** | (Gyori, 2023) | (Robb et al., 2020; Wister, Li, Levasseur, et al., 2023) | (van den Besselaar et al., 2021) |
| **Others** |  |  |  |
| Social isolation/ less social contact/Loneliness/less internet use | (Creese et al., 2021; Holwerda et al., 2023; Robb et al., 2020) |  |  |
| Neuroticism(Personality) | (Holwerda et al., 2023) |  |  |
| Coping strategy/Praying |  |  | (Holwerda et al., 2023) |
| Mastery |  |  | (Holwerda et al., 2023) |
| Poor sleep | (Robb et al., 2020) |  |  |
| Hopelessness | (Amerio et al., 2023) |  |  |
