# Supplementary material for: Changes in mental health outcomes of middle-aged and older adults during the COVID-19 pandemic: A scoping review
Source: PLoS One. 2026 Aug 27;21(8):e0342140. doi: 10.1371/journal.pone.0342140 (PMC13521375; doi:10.1371/journal.pone.0342140)
Supplement: Supplementary file 5 [file pone.0342140.s006.docx]

S3 Table. Factors associated with change in Loneliness (n=15 studies)

| Factors | Positive- Increase in symptoms | Negative: Decrease in symptoms | Not Significant |
| --- | --- | --- | --- |
| **Socio-demographics** |  |  |  |
| Age | (Arpino et al., 2022; Gyori, 2023; Holwerda et al., 2023) | (Kirkland et al., 2023) | (Dhakal et al., 2023; Fuller & Huseth-Zosel, 2022; Wester et al., 2022; Zaninotto et al., 2022) |
| Sex: Female | (Arpino et al., 2022; Dhakal et al., 2023; Gyori, 2023; Kirkland et al., 2023; Wester et al., 2022; Zaninotto et al., 2022) | (Holwerda et al., 2023) | (Fuller & Huseth-Zosel, 2022; Richardson et al., 2023) |
| Ethnicity: White |  | (Dhakal et al., 2023) | (Fuller & Huseth-Zosel, 2022; Kirkland et al., 2023) |
| Marital Status:Not married | (Zaninotto et al., 2022) | (Gyori, 2023) | (Dhakal et al., 2023; Holwerda et al., 2023) |
| Rural/ Village |  | (Fuller & Huseth-Zosel, 2022; Kirkland et al., 2023) |  |
| **Socio-economic** |  |  |  |
| Education: Higher education | (Fuller & Huseth-Zosel, 2022; Kirkland et al., 2023) | (Gyori, 2023) | (Dhakal et al., 2023; Holwerda et al., 2023; Wester et al., 2022) |
| Digital literacy: Yes |  | (Dhakal et al., 2023) |  |
| Employment: Unemployed | (Arpino et al., 2022; Fuller & Huseth-Zosel, 2022; Gyori, 2023) |  | (Wester et al., 2022) |
| Income: Less | (Dhakal et al., 2023) | (Zaninotto et al., 2022) | (Holwerda et al., 2023) |
| **Health** |  |  | (Kirkland et al., 2023) |
| Comorbidities | (Arpino et al., 2022; Gyori, 2023; Kirkland et al., 2023) |  |  |
| Self-rated health: poor | (Richardson et al., 2023) |  |  |
| Disability |  |  |  |
| Pre-Covid mental health | (Holwerda et al., 2023) |  |  |
| **COVID-19 related factors** |  |  |  |
| Infection status | (Richardson et al., 2023; Wester et al., 2022) |  |  |
| Stringency index: Low |  | (Wester et al., 2022) |  |
| COVID-19 exposure | (Holwerda et al., 2023) |  | (Gyori, 2023) |
| Vaccination | (Holwerda et al., 2023) |  |  |
| **Behavioral factors** |  |  |  |
| Alcohol: No drinker |  | (Kirkland et al., 2023) |  |
| Smoking: No smoker |  |  | (Kirkland et al., 2023) |
| Physical Activity(ADL/IADL/Frality): Limited activity | (Arpino et al., 2022; Holwerda et al., 2023) |  | (Kirkland et al., 2023; Wester et al., 2022) |
| **Living Situation-Living Alone** | (Arpino et al., 2022; Dhakal et al., 2023; Gyori, 2023; Kirkland et al., 2023; L et al., 2022; Richardson et al., 2023; Stolz et al., 2021; Wester et al., 2022) |  | (Fuller & Huseth-Zosel, 2022) |
| **Others** |  |  |  |
| Social isolation/ less social contact | (Gyori, 2023; Holwerda et al., 2023; Kirkland et al., 2023; L et al., 2022; Richardson et al., 2023) |  |  |
| Housetype |  |  | (Kirkland et al., 2023) |
| Depression | (Dhakal et al., 2023; Kirkland et al., 2023; L et al., 2022) |  |  |
| Mastery | (Holwerda et al., 2023) |  |  |
| Neuroticism(Personality) | (Holwerda et al., 2023) |  |  |
| Coping strategy/Praying |  |  | (Holwerda et al., 2023) |
