# Supplementary material for: Changes in mental health outcomes of middle-aged and older adults during the COVID-19 pandemic: A scoping review
Source: PLoS One. 2026 Aug 27;21(8):e0342140. doi: 10.1371/journal.pone.0342140 (PMC13521375; doi:10.1371/journal.pone.0342140)
Supplement: Supplementary file 6 [file pone.0342140.s008.docx]

Response to Reviewer and Editor Comments

We would like to thank both the reviewer and the academic editor for their careful evaluation of our manuscript and their valuable feedback and comments. We have carefully considered all suggestions and have revised the manuscript accordingly. Please find our response to comments and changes made in the manuscript as follows.

**Response to Academic Editor comments**

1. Please ensure that your manuscript meets PLOS ONE's style requirements, including those for file naming.

Author response*: We reviewed all the instructions provided and ensured that our manuscript fully meets the criteria.*

2. Please ensure that you have uploaded your PRISMA flowchart as Figure 1, or else explain why this is not possible

Author response: *We have revised the PRISMA flowchart in accordance with the format provided in the email. It is now labeled as Fig 1*.

Please complete your Competing Interests on the online submission form to state any Competing Interests. If you have no competing interests, please state "The authors have declared that no competing interests exist," as detailed online in our guide for authors. This information should be included in your cover letter; we will change the online submission form on your behalf.

Author response: *We have stated “The authors have declared that no competing interests exist” in both the manuscript and the cover letter.*

4.Please provide a complete Data Availability Statement in the submission form, ensuring you include all necessary access information or a reason why you are unable to make your data freely accessible. If your research concerns only data provided within your submission, please write "All data are in the manuscript and/or supporting information files" as your Data Availability Statement.

Author response: *All data are in the manuscript and/or supporting information files is written in submission form.*

5. PLOS requires an ORCID iD for the corresponding author in Editorial Manager on papers submitted after December 6th, 2016. Please ensure that you have an ORCID iD and that it is validated in Editorial Manager.

Author response: *ORCID id have been validated.*

*6.* Please review your reference list to ensure that it is complete and correct. If you have cited papers that have been retracted, please include the rationale for doing so in the manuscript text, or remove these references and replace them with relevant current references

Author: *We have checked all the references. We have added one citation as suggested by the reviewer. We reviewed the reference and it is relevant to our topic.*

(5) Silva C, Ferreira R, Morgado B, Alves E, Fonseca C. Depression, Loneliness and Quality of Life in Institutionalized and Non-Institutionalized Older Adults in Portugal: A Cross-Sectional Study. Nursing Reports 2024, Vol 14, Pages 2340-2354. 2024 Sep 10;14(3):2340–54. doi:10.3390/nursrep14030174” .

*Also, we have updated the reference for the news article in correct format.* The original citation:

Moreno-Agostino D, MC, ORC, & ME. Pandemic may have triggered second “midlife crisis” for over-50s. Kings College London. 2023. Available from: https://www.kcl.ac.uk/news/pandemic-triggered-second-midlife-crisis-for-over-50s

To:.	Pandemic may have triggered second “midlife crisis” for over-50s | King’s College London [Internet]. [cited 2026 Mar 24]. Available from: https://www.kcl.ac.uk/news/pandemic-triggered-second-midlife-crisis-for-over-50s

(9) *In addition, we have now cited the original research paper underlying this news report to ensure proper citation of the findings.*

(8) Moreno-Agostino D, Fisher HL, Goodman A, Hatch SL, Morgan C, Richards M, et al. Long-term psychological distress trajectories and the COVID-19 pandemic in three British birth cohorts: A multi-cohort study. PLoS Med. 2023 Apr 1;20(4). doi:10.1371/journal.pmed.1004145 PubMed PMID: 37014820.

**Response to reviewer questions**

**Comment 1:**
At the end of the “Abstract” section, it is stated that “monitoring mental health outcomes is essential to track long-term changes and to prepare for future public health emergencies”. This statement is repeated in the conclusion. Although some repetition is acceptable between the abstract and conclusion, the language could be differentiated to increase the impact. I suggest that the authors slightly rephrase the concluding sentence to emphasize a different aspect, such as specific health policy recommendations.

Author response:

*We have change the sentence in conclusion from* “These research gaps represent a significant limitation in our understanding of the broader contextual mental health risk factors during the COVID-19 pandemic among middle-aged and older adults, underscoring the need for continued investigation to fully understand the long-term effects of COVID-19 on mental health among middle-aged and older adults and better prepare for future public health crises. “ *to* “These research gaps represent a significant limitation in our understanding of the broader contextual mental health trend and risk factors during the COVID-19 pandemic among middle-aged and older adults, underscoring the need for continued investigation to guide policy development and form targeted strategies for better understanding, preparedness and managing of adverse mental health outcome among middle age and older adults during any future public health “ on line 436 to 442.

Comment 2:
The ‘Introduction’ section is well written and frames the topic and its relevance very well. In lines 76-78, the statement ‘While much attention was given to infection rates and mortality, the pandemic also brought about significant challenges to mental health as people faced rapid changes, uncertainty, and social isolation [4]’ is very pertinent. I suggest that the authors consider adding the following: ‘While much attention was given to infection rates and mortality, the pandemic also brought about significant challenges to mental health as people faced rapid changes, uncertainty, and social isolation [4], changes that have persisted into the post-COVID period [5]’.
[5] - Silva, C., Ferreira, R., Morgado, B., Alves, E., & Fonseca, C. (2024). Depression, loneliness and quality of life in institutionalised and non-institutionalised older adults in Portugal: A cross-sectional study. Nursing Reports, 14(3), 2340-2354. DOI:

Author response:

We have reviewed the citation and found it important, and thus added the citation and reframe the sentence as “While much attention was given to infection rates and mortality, the pandemic also brought about significant challenges to mental health as people faced rapid changes, uncertainty, and social isolation [4], many of which persisted into the post-COVID period [5]”. On line 77, 78

Comment 3:

In the section ‘Changes in mental health outcomes’, subsection ‘Depressive symptoms’, lines 212-214, it is stated that ‘Seventeen out of 23 studies that reported changes in depressive symptoms’.
However, Table 1 indicates that 30 studies measured depressive symptoms (68.2% of 44). This discrepancy of 30 vs. 23 is not explained. It is unclear whether the remaining 7 studies did not report changes, were excluded from some sub-analysis, or whether there is a counting error.
I suggest that the authors explain the discrepancy between the total number of studies with depression (n=30 in Table 1) and the studies used in the narrative synthesis of changes (n=23). If some studies measured only point prevalence and not change, this should be explicitly clarified in the methods.

Author response:

*We have revised the text on lines 207–209 for clarity. The original wording has been changed to*:

“Twenty-three studies out of 30 studies reported changes in depressive symptoms during the global spread and response phase, compared to pre-pandemic levels or the initial outbreak phase, with 17 out of 23 indicating an increase in depressive symptoms.”

This revision clarifies the number of studies included and clearly describes the observed changes in depressive symptoms across the different pandemic phases.

**Comment 4:**
Reference 6 is “Moreno-Agostino D, MC, ORC, & ME. Pandemic may have triggered second ‘midlife crisis’ for over-50s. Kings College London. 2023.” It appears that the names of the co-authors are missing. I suggest that the authors review whether this reference is correct.

Author response: The reference have been corrected and updated as mentioned above.

**Comment 5:**
Figure 3 uses overlapping circles to illustrate risk factors by mental health domain, but the circles have partially cut-off text. I suggest that the authors correct the layout of Figure 3 to ensure that all text is legible.

Author response: We have edited the figure 3 as suggested.
