# Supplementary material for: Changes in mental health outcomes of middle-aged and older adults during the COVID-19 pandemic: A scoping review
Source: PLoS One. 2026 Aug 27;21(8):e0342140. doi: 10.1371/journal.pone.0342140 (PMC13521375; doi:10.1371/journal.pone.0342140)
Supplement: Supplementary file 7 [file pone.0342140.s009.docx]

**Response to Reviewer Comments**

We would like to thank the reviewers for their careful evaluation of our manuscript and their valuable feedback and comments. We have considered all suggestions and have revised the manuscript accordingly. Please find our response to the comments and changes made in the manuscript as follows.

**Response to reviewer comments:**

**Comment 1: Better justify the eligibility criterion “well-being during the COVID-19 pandemic using the same scales for comparability.”**

*Author response*: We have added the following for the justification: “This criterion was applied to ensure that observed changes in mental health outcomes were attributable to changes in mental health status rather than variations in measurement resulting from the use of different scales.” On lines 154- 156

**Comment 2: “Explicitly acknowledge that no assessment of the methodological quality (risk of bias) of the included studies was performed”.**
*Author response:* We have added “No review protocol was registered, and no formal assessment of methodological quality (risk of bias) of the included studies was performed [18]” in the methods section, on line 124 to 126

**Comment 3: The substantial heterogeneity among the included studies (e.g., countries, follow-up periods, measurement instruments, and study designs) should also be discussed in greater depth, as it limits the comparability of the results.**

**And finally, the “Discussion” section should present a more critical interpretation of the results, rather than limiting itself primarily to summarizing the evidence. In particular, the authors should discuss possible explanations for the heterogeneity among the studies**

*Author response*: Thank you for the comment. We had mentioned the limitation in terms of periods and measurement in the discussion section: “Another significant limitation of this scoping review is the inclusion of studies employing varied diagnostic tools and effect estimates, which complicates the synthesis of findings and may obscure the overall interpretability and comparability of the results across studies. Studies included in this review exhibit significant variability in both pre-pandemic measurement periods and follow-up periods, affecting cross-study comparability. For instance, a study by Besselaar and colleagues used data from 2011-2019 as a pre-pandemic baseline [28], whereas Amerio and colleagues relied on 2019 data for baseline measures [19], which may affect the comparability across studies.” On lines 440 to 455 of the main manuscript.

We now address the heterogeneity across findings in terms of countries and design by adding the following paragraph (Line 367 to 385 ) to the discussion section: “The substantial heterogeneity in results may be attributable to differences in countries and study designs. Across countries, increases in mental health problems were reported in many settings, including Canada, the United Kingdom, Germany, Japan, Korea, the Faroe Islands, Bangladesh, the Netherlands, Austria, Finland, Chile, and Italy. For example, the majority of Canadian, European, and Asian (excluding China) studies reported worsening mental health outcomes [19–21,23,24,26,28,33,42,44,48,50]. In contrast, studies from the United States and China showed more heterogeneous findings, with some reporting increases [32,51,53,55,58,59], and others reporting decreases or no significant changes [34,36,38,39,45,46]. Outside this region, the study from Brazil reported a decrease in loneliness during the pandemic period [62], and New Zealand showed no significant change [40]. Differences in study design may also explain variation in findings. Most longitudinal studies reported increases in mental health symptoms [20,21,24,26,32,33,42,44,50,55]; however, some studies observed stable or decreased symptoms [34,36,38–40,45,46,62]. In contrast, all included cross-sectional studies showed increases in mental health symptoms during the pandemic; however, interpretation is generally limited because these studies often rely on comparisons with recalled pre-pandemic status [19,23,28,31,43,48,51,53,54]. Overall, the direction and magnitude of changes were not uniform across countries or study designs. The variation suggests that the impact of the pandemic on mental health was influenced not only by the occurrence of COVID-19 itself but also by contextual factors like countries and study design.”

**Comment 4*:* Throughout the manuscript, use the term “risk factors” with caution. It is appropriate only when discussing longitudinal studies that actually assess true risk. When describing the results of cross-sectional studies, replace it with “factors associated with changes in mental health outcomes,” as these studies do not support causal inferences.**
*Author response:* Thank you for bringing this to our attention. The paper summarizes results from a pooled body of both cross-sectional and longitudinal studies, and even for longitudinal studies we cannot guarantee causal inference. Accordingly, we have revised the manuscript to replace “risk factors” with “associated factors” throughout, as our intention in this scoping review is not to establish causal inferences but to identify associated factors.

**Comment 5: The “Methods” section would also benefit from clarifying whether a review protocol was registered.**

*Author response:* We have added: “No review protocol was registered, and no formal assessment of methodological quality (risk of bias) of the included studies was performed [18]” in the methods section on line on line 124 to 126

We would once again like to thank the reviewers for their helpful comments and suggestions. These revisions have strengthened our manuscript.

If you require any further revisions or clarifications, please do not hesitate to contact us.
