# Supplementary material for: Changes in mental health outcomes of middle-aged and older adults during the COVID-19 pandemic: A scoping review
Source: PLoS One. 2026 Aug 27;21(8):e0342140. doi: 10.1371/journal.pone.0342140 (PMC13521375; doi:10.1371/journal.pone.0342140)
Supplement: Peer review history

# Peer review: Decision Letter 0

**DOI:** 10.1371/journal.pone.0342140.r001

**Authors:** Ilse Bloom

17 Mar 2026

Dear Dr. Wilk,

Please note that we have only been able to secure a single reviewer to assess your manuscript. We are issuing a decision on your manuscript at this point to prevent further delays in the evaluation of your manuscript. Please be aware that the editor who handles your revised manuscript might find it necessary to invite additional reviewers to assess this work once the revised manuscript is submitted. However, we will aim to proceed on the basis of this single review if possible.

The comments from the reviewer can be found below. Please review these comments and make the appropriate revisions to your manuscript alongside a point-by-point response to the concerns raised.

Please submit your revised manuscript by May 01 2026 11:59PM. If you will need more time than this to complete your revisions, please reply to this message or contact the journal office at plosone@plos.org. When you're ready to submit your revision, log on to https://www.editorialmanager.com/pone/ and select the 'Submissions Needing Revision' folder to locate your manuscript file.

- A letter that responds to each point raised by the academic editor and reviewer(s). You should upload this letter as a separate file labeled 'Response to Reviewers'.
- A marked-up copy of your manuscript that highlights changes made to the original version. You should upload this as a separate file labeled 'Revised Manuscript with Track Changes'.
- An unmarked version of your revised paper without tracked changes. You should upload this as a separate file labeled 'Manuscript'.

If you would like to make changes to your financial disclosure, please include your updated statement in your cover letter. Guidelines for resubmitting your figure files are available below the reviewer comments at the end of this letter.

https://journals.plos.org/plosone/s/submission-guidelines#loc-laboratory-protocols. Additionally, PLOS ONE offers an option for publishing peer-reviewed Lab Protocol articles, which describe protocols hosted on protocols.io. Read more information on sharing protocols at https://plos.org/protocols?utm_medium=editorial-email&utm_source=authorletters&utm_campaign=protocols.

We look forward to receiving your revised manuscript.

Kind regards,

Ilse Bloom

Staff Editor

PLOS One

Journal Requirements:

When submitting your revision, we need you to address these additional requirements.

1. Please ensure that your manuscript meets PLOS ONE's style requirements, including those for file naming. The PLOS ONE style templates can be found at

https://journals.plos.org/plosone/s/file?id=wjVg/PLOSOne_formatting_sample_main_body.pdf and https://journals.plos.org/plosone/s/file?id=ba62/PLOSOne_formatting_sample_title_authors_affiliations.pdf

2. Please ensure that you have uploaded your PRISMA flowchart as Figure 1, or else explain why this is not possible. Blank flowcharts can be found here: http://www.prisma-statement.org/

3. Thank you for stating the following in your Competing Interests section:

[We declare no conflicting interest].

Please complete your Competing Interests on the online submission form to state any Competing Interests. If you have no competing interests, please state "The authors have declared that no competing interests exist.", as detailed online in our guide for authors at http://journals.plos.org/plosone/s/submit-now

This information should be included in your cover letter; we will change the online submission form on your behalf.

4. Please provide a complete Data Availability Statement in the submission form, ensuring you include all necessary access information or a reason for why you are unable to make your data freely accessible. If your research concerns only data provided within your submission, please write "All data are in the manuscript and/or supporting information files" as your Data Availability Statement.

5. PLOS requires an ORCID iD for the corresponding author in Editorial Manager on papers submitted after December 6th, 2016. Please ensure that you have an ORCID iD and that it is validated in Editorial Manager. To do this, go to ‘Update my Information’ (in the upper left-hand corner of the main menu), and click on the Fetch/Validate link next to the ORCID field. This will take you to the ORCID site and allow you to create a new iD or authenticate a pre-existing iD in Editorial Manager.

If the reviewer comments include a recommendation to cite specific previously published works, please review and evaluate these publications to determine whether they are relevant and should be cited. There is no requirement to cite these works unless the editor has indicated otherwise.

Please review your reference list to ensure that it is complete and correct. If you have cited papers that have been retracted, please include the rationale for doing so in the manuscript text, or remove these references and replace them with relevant current references. Any changes to the reference list should be mentioned in the rebuttal letter that accompanies your revised manuscript. If you need to cite a retracted article, indicate the article’s retracted status in the References list and also include a citation and full reference for the retraction notice.

[Note: HTML markup is below. Please do not edit.]

Reviewers' comments:

Reviewer's Responses to Questions

**Comments to the Author**

1. Is the manuscript technically sound, and do the data support the conclusions?

Reviewer #1: Yes

**********

2. Has the statistical analysis been performed appropriately and rigorously?

Reviewer #1: N/A

**********

3. Have the authors made all data underlying the findings in their manuscript fully available?

The PLOS Data policy

Reviewer #1: Yes

**********

4. Is the manuscript presented in an intelligible fashion and written in standard English?

Reviewer #1: Yes

**********

Reviewer #1: Dear authors,

Thank you very much for the opportunity to review this manuscript.

The topic addressed is very relevant and, if published, has great potential to be cited.

The methodology seems appropriate to me and the text is clear to the reader.

This is a methodically structured scoping review that addresses a relevant public health issue: changes in mental health outcomes in middle-aged and older adults during the COVID-19 pandemic. The work has scientific merit, especially for its focus on changes (rather than just prevalence) and its coverage of multiple domains of mental health throughout the three phases of the pandemic.

The manuscript seems to me to be very well written.

I have only five comments to make in order to strengthen the manuscript.

Comment 1:

At the end of the “Abstract” section, it is stated that “monitoring mental health outcomes is essential to track long-term changes and to prepare for future public health emergencies”. This statement is repeated in the conclusion. Although some repetition is acceptable between the abstract and conclusion, the language could be differentiated to increase the impact. I suggest that the authors slightly rephrase the sentence in the conclusion to emphasise a different aspect, such as specific health policy recommendations.

Comment 2:

The ‘Introduction’ section is well written and frames the topic and its relevance very well. In lines 76-78, the statement ‘While much attention was given to infection rates and mortality, the pandemic also brought about significant challenges to mental health as people faced rapid changes, uncertainty, and social isolation [4]’ is very pertinent. I suggest that the authors consider adding the following: ‘While much attention was given to infection rates and mortality, the pandemic also brought about significant challenges to mental health as people faced rapid changes, uncertainty, and social isolation [4], changes that have persisted into the post-COVID period [5]’.

[5] - Silva, C., Ferreira, R., Morgado, B., Alves, E., & Fonseca, C. (2024). Depression, loneliness and quality of life in institutionalised and non-institutionalised older adults in Portugal: A cross-sectional study. Nursing Reports, 14(3), 2340-2354. DOI: https://doi.org/10.3390/nursrep14030174

Comment 3:

In the section ‘Changes in mental health outcomes’, subsection ‘Depressive symptoms’, lines 212-214, it is stated that ‘Seventeen out of 23 studies that reported changes in depressive symptoms’.

However, Table 1 indicates that 30 studies measured depressive symptoms (68.2% of 44). This discrepancy of 30 vs. 23 is not explained. It is unclear whether the remaining 7 studies did not report changes, were excluded from some sub-analysis, or whether there is a counting error.

I suggest that the authors explain the discrepancy between the total number of studies with depression (n=30 in Table 1) and the studies used in the narrative synthesis of changes (n=23). If some studies measured only point prevalence and not change, this should be explicitly clarified in the methods.

Comment 4:

Reference 6 is “Moreno-Agostino D, MC, ORC, & ME. Pandemic may have triggered second ‘midlife crisis’ for over-50s. Kings College London. 2023.” It appears that the names of the co-authors are missing. I suggest that the authors review whether this reference is correct.

Comment 5:

Figure 3 uses overlapping circles to illustrate risk factors by mental health domain, but the circles have partially cut-off text. I suggest that the authors correct the layout of Figure 3 to ensure that all text is legible.

Kind regards.

**********

what does this mean?). If published, this will include your full peer review and any attached files.

If you choose “no”, your identity will remain anonymous but your review may still be made public.

**Do you want your identity to be public for this peer review?**  For information about this choice, including consent withdrawal, please see our Privacy Policy

Reviewer #1: No

**********

[NOTE: If reviewer comments were submitted as an attachment file, they will be attached to this email and accessible via the submission site. Please log into your account, locate the manuscript record, and check for the action link "View Attachments". If this link does not appear, there are no attachment files.]

To ensure your figures meet our technical requirements, please review our figure guidelines: https://journals.plos.org/plosone/s/figures

You may also use PLOS’s free figure tool, NAAS, to help you prepare publication quality figures: https://journals.plos.org/plosone/s/figures#loc-tools-for-figure-preparation.

NAAS will assess whether your figures meet our technical requirements by comparing each figure against our figure specifications.

# Author comment: Author response to Decision Letter 1

**DOI:** 10.1371/journal.pone.0342140.r002

2 May 2026

Response to Reviewer and Editor Comments

We would like to thank both the reviewer and the academic editor for their careful evaluation of our manuscript and their valuable feedback and comments. We have carefully considered all suggestions and have revised the manuscript accordingly. Please find our response to comments and changes made in the manuscript as follows.

Response to Academic Editor comments

1. Please ensure that your manuscript meets PLOS ONE's style requirements, including those for file naming.

Author response: We reviewed all the instructions provided and ensured that our manuscript fully meets the criteria.

2. Please ensure that you have uploaded your PRISMA flowchart as Figure 1, or else explain why this is not possible

Author response: We have revised the PRISMA flowchart in accordance with the format provided in the email. It is now labeled as Fig 1.

Please complete your Competing Interests on the online submission form to state any Competing Interests. If you have no competing interests, please state "The authors have declared that no competing interests exist," as detailed online in our guide for authors. This information should be included in your cover letter; we will change the online submission form on your behalf.

Author response: We have stated “The authors have declared that no competing interests exist” in both the manuscript and the cover letter.

4.Please provide a complete Data Availability Statement in the submission form, ensuring you include all necessary access information or a reason why you are unable to make your data freely accessible. If your research concerns only data provided within your submission, please write "All data are in the manuscript and/or supporting information files" as your Data Availability Statement.

Author response: All data are in the manuscript and/or supporting information files is written in submission form.

5. PLOS requires an ORCID iD for the corresponding author in Editorial Manager on papers submitted after December 6th, 2016. Please ensure that you have an ORCID iD and that it is validated in Editorial Manager.

Author response: ORCID id have been validated.

6. Please review your reference list to ensure that it is complete and correct. If you have cited papers that have been retracted, please include the rationale for doing so in the manuscript text, or remove these references and replace them with relevant current references

Author: We have checked all the references. We have added one citation as suggested by the reviewer. We reviewed the reference and it is relevant to our topic.

(5) Silva C, Ferreira R, Morgado B, Alves E, Fonseca C. Depression, Loneliness and Quality of Life in Institutionalized and Non-Institutionalized Older Adults in Portugal: A Cross-Sectional Study. Nursing Reports 2024, Vol 14, Pages 2340-2354. 2024 Sep 10;14(3):2340–54. doi:10.3390/nursrep14030174” .

Also, we have updated the reference for the news article in correct format. The original citation:

Moreno-Agostino D, MC, ORC, & ME. Pandemic may have triggered second “midlife crisis” for over-50s. Kings College London. 2023. Available from: https://www.kcl.ac.uk/news/pandemic-triggered-second-midlife-crisis-for-over-50s

To:. Pandemic may have triggered second “midlife crisis” for over-50s | King’s College London [Internet]. [cited 2026 Mar 24]. Available from: https://www.kcl.ac.uk/news/pandemic-triggered-second-midlife-crisis-for-over-50s

(9) In addition, we have now cited the original research paper underlying this news report to ensure proper citation of the findings.

(8) Moreno-Agostino D, Fisher HL, Goodman A, Hatch SL, Morgan C, Richards M, et al. Long-term psychological distress trajectories and the COVID-19 pandemic in three British birth cohorts: A multi-cohort study. PLoS Med. 2023 Apr 1;20(4). doi:10.1371/journal.pmed.1004145 PubMed PMID: 37014820.

Response to reviewer questions

Comment 1:

At the end of the “Abstract” section, it is stated that “monitoring mental health outcomes is essential to track long-term changes and to prepare for future public health emergencies”. This statement is repeated in the conclusion. Although some repetition is acceptable between the abstract and conclusion, the language could be differentiated to increase the impact. I suggest that the authors slightly rephrase the concluding sentence to emphasize a different aspect, such as specific health policy recommendations.

Author response:

We have change the sentence in conclusion from “These research gaps represent a significant limitation in our understanding of the broader contextual mental health risk factors during the COVID-19 pandemic among middle-aged and older adults, underscoring the need for continued investigation to fully understand the long-term effects of COVID-19 on mental health among middle-aged and older adults and better prepare for future public health crises. “ to “These research gaps represent a significant limitation in our understanding of the broader contextual mental health trend and risk factors during the COVID-19 pandemic among middle-aged and older adults, underscoring the need for continued investigation to guide policy development and form targeted strategies for better understanding, preparedness and managing of adverse mental health outcome among middle age and older adults during any future public health “ on line 436 to 442.

Comment 2:

The ‘Introduction’ section is well written and frames the topic and its relevance very well. In lines 76-78, the statement ‘While much attention was given to infection rates and mortality, the pandemic also brought about significant challenges to mental health as people faced rapid changes, uncertainty, and social isolation [4]’ is very pertinent. I suggest that the authors consider adding the following: ‘While much attention was given to infection rates and mortality, the pandemic also brought about significant challenges to mental health as people faced rapid changes, uncertainty, and social isolation [4], changes that have persisted into the post-COVID period [5]’.

[5] - Silva, C., Ferreira, R., Morgado, B., Alves, E., & Fonseca, C. (2024). Depression, loneliness and quality of life in institutionalised and non-institutionalised older adults in Portugal: A cross-sectional study. Nursing Reports, 14(3), 2340-2354. DOI:

Author response:

We have reviewed the citation and found it important, and thus added the citation and reframe the sentence as “While much attention was given to infection rates and mortality, the pandemic also brought about significant challenges to mental health as people faced rapid changes, uncertainty, and social isolation [4], many of which persisted into the post-COVID period [5]”. On line 77, 78

Comment 3:

In the section ‘Changes in mental health outcomes’, subsection ‘Depressive symptoms’, lines 212-214, it is stated that ‘Seventeen out of 23 studies that reported changes in depressive symptoms’.

However, Table 1 indicates that 30 studies measured depressive symptoms (68.2% of 44). This discrepancy of 30 vs. 23 is not explained. It is unclear whether the remaining 7 studies did not report changes, were excluded from some sub-analysis, or whether there is a counting error.

I suggest that the authors explain the discrepancy between the total number of studies with depression (n=30 in Table 1) and the studies used in the narrative synthesis of changes (n=23). If some studies measured only point prevalence and not change, this should be explicitly clarified in the methods.

Author response:

We have revised the text on lines 207–209 for clarity. The original wording has been changed to:

“Twenty-three studies out of 30 studies reported changes in depressive symptoms during the global spread and response phase, compared to pre-pandemic levels or the initial outbreak phase, with 17 out of 23 indicating an increase in depressive symptoms.”

This revision clarifies the number of studies included and clearly describes the observed changes in depressive symptoms across the different pandemic phases.

Comment 4:

Reference 6 is “Moreno-Agostino D, MC, ORC, & ME. Pandemic may have triggered second ‘midlife crisis’ for over-50s. Kings College London. 2023.” It appears that the names of the co-authors are missing. I suggest that the authors review whether this reference is correct.

Author response: The reference have been corrected and updated as mentioned above.

Comment 5:

Figure 3 uses overlapping circles to illustrate risk factors by mental health domain, but the circles have partially cut-off text. I suggest that the authors correct the layout of Figure 3 to ensure that all text is legible.

Author response: We have edited the figure 3 as suggested.

4.Please provide a complete Data Availability Statement in the submission form, ensuring you include all necessary access information or a reason why you are unable to make your data freely accessible. If your research concerns only data provided within your submission, please write "All data are in the manuscript and/or supporting information files" as your Data Availability Statement.

Author response: All data are in the manuscript and/or supporting information files is written in submission form.

5. PLOS requires an ORCID iD for the corresponding author in Editorial Manager on papers submitted after December 6th, 2016. Please ensure that you have an ORCID iD and that it is validated in Editorial Manager.

Author response: ORCID id have been validated.

6. Please review your reference list to ensure that it is complete and correct. If you have cited papers that have been retracted, please include the rationale for doing so in the manuscript text, or remove these references and replace them with relevant current references

Author: We have checked all the references. We have added one citation as suggested by the reviewer. We reviewed the reference and it is relevant to our topic.

(5) Silva C, Ferreira R, Morgado B, Alves E, Fonseca C. Depression, Loneliness and Quality of Life in Institutionalized and Non-Institutionalized Older Adults in Portugal: A Cross-Sectional Study. Nursing Reports 2024, Vol 14, Pages 2340-2354. 2024 Sep 10;14(3):2340–54. doi:10.3390/nursrep14030174” .

Also, we have updated the reference for the news article in correct format. The original citation:

Moreno-Agostino D, MC, ORC, & ME. Pandemic may have triggered second “midlife crisis” for over-50s. Kings College London. 2023. Available from: https://www.kcl.ac.uk/news/pandemic-triggered-second-midlife-crisis-for-over-50s

To:. Pandemic may have triggered second “midlife crisis” for over-50s | King’s College London [Internet]. [cited 2026 Mar 24]. Available from: https://www.kcl.ac.uk/news/pandemic-triggered-second-midlife-crisis-for-over-50s

(9) In addition, we have now cited the original research paper underlying this news report to ensure proper citation of the findings.

(8) Moreno-Agostino D, Fisher HL, Goodman A, Hatch SL, Morgan C, Richards M, et al. Long-term psychological distress trajectories and the COVID-19 pandemic in three British birth cohorts: A multi-cohort study. PLoS Med. 2023 Apr 1;20(4). doi:10.1371/journal.pmed.1004145 PubMed PMID: 37014820.

Response to reviewer questions

Comment 1:

At the end of the “Abstract” section, it is stated that “monitoring mental health outcomes is essential to track long-term changes and to prepare for future public health emergencies”. This statement is repeated in the conclusion. Although some repetition is acceptable between the abstract and conclusion, the language could be differentiated to increase the impact. I suggest that the authors slightly rephrase the concluding sentence to emphasize a different aspect, such as specific health policy recommendations.

Author response:

We have change the sentence in conclusion from “These research gaps represent a significant limitation in our understanding of the broader contextual mental health risk factors during the COVID-19 pandemic among middle-aged and older adults, underscoring the need for continued investigation to fully understand the long-term effects of COVID-19 on mental health among middle-aged and older adults and better prepare for future public health crises. “ to “These research gaps represent a significant limitation in our understanding of the broader contextual mental health trend and risk factors during the COVID-19 pandemic among middle-aged and older adults, underscoring the need for continued investigation to guide policy development and form targeted strategies for better understanding, preparedness and managing of adverse mental health outcome among middle age and older adults during any future public health “ on line 432 to 438

Comment 2:

The ‘Introduction’ section is well written and frames the topic and its relevance very well. In lines 76-78, the statement ‘While much attention was given to infection rates and mortality, the pandemic also brought about significant challenges to mental health as people faced rapid changes, uncertainty, and social isolation [4]’ is very pertinent. I suggest that the authors consider adding the following: ‘While much attention was given to infection rates and mortality, the pandemic also brought about significant challenges to mental health as people faced rapid changes, uncertainty, and social isolation [4], changes that have persisted into the post-COVID period [5]’.

[5] - Silva, C., Ferreira, R., Morgado, B., Alves, E., & Fonseca, C. (2024). Depression, loneliness and quality of life in institutionalised and non-institutionalised older adults in Portugal: A cross-sectional study. Nursing Reports, 14(3), 2340-2354. DOI:

Author response:

We have reviewed the citation and found it important, and thus added the citation and reframe the sentence as “While much attention was given to infection rates and mortality, the pandemic also brought about significant challenges to mental health as people faced rapid changes, uncertainty, and social isolation [4], many of which persisted into the post-COVID period [5]”. On line 77

Comment 3:

In the section ‘Changes in mental health outcomes’, subsection ‘Depressive symptoms’, lines 212-214, it is stated that ‘Seventeen out of 23 studies that reported changes in depressive symptoms’.

However, Table 1 indicates that 30 studies measured depressive symptoms (68.2% of 44). This discrepancy of 30 vs. 23 is not explained. It is unclear whether the remaining 7 studies did not report changes, were excluded from some sub-analysis, or whether there is a counting error.

I suggest that the authors explain the discrepancy between the total number of studies with depression (n=30 in Table 1) and the studies used in the narrative synthesis of changes (n=23). If some studies measured only point prevalence and not change, this should be explicitly clarified in the methods.

Author response:

We have revised the text on lines 208–210 for clarity. The original wording has been changed to:

“Twenty-three studies out of 30 studies reported changes in depressive symptoms during the global spread and response phase, compared to pre-pandemic levels or the initial outbreak phase, with 17 out of 23 indicating an increase in depressive symptoms.”

This revision clarifies the number of studies included and clearly describes the observed changes in depressive symptoms across the different pandemic phases.

Comment 4:

Reference 6 is “Moreno-Agostino D, MC, ORC, & ME. Pandemic may have triggered second ‘midlife crisis’ for over-50s. Kings College London. 2023.” It appears that the names of the co-authors are missing. I suggest that the authors review whether this reference is correct.

Author response: The reference have been corrected and updated as mentioned above.

Comment 5:

Figure 3 uses overlapping circles to illustrate risk factors by mental health domain, but the circles have partially cut-off text. I suggest that the authors correct the layout of Figure 3 to ensure that all text is legible.

Author response: We have edited the figure 3 as suggested

Attachment Submitted filename: Response to Reviewers.docx

# Peer review: Decision Letter 1

**DOI:** 10.1371/journal.pone.0342140.r003

**Authors:** Jiaci Lin

26 Jul 2026

Dear Dr. Wilk,

Thank you for submitting your manuscript to PLOS ONE. After careful consideration, we feel that it has merit but does not fully meet PLOS ONE’s publication criteria as it currently stands. Therefore, we invite you to submit a revised version of the manuscript that addresses the points raised during the review process.

Please submit your revised manuscript by Sep 09 2026 11:59PM. If you will need more time than this to complete your revisions, please reply to this message or contact the journal office at plosone@plos.org. When you're ready to submit your revision, log on to https://www.editorialmanager.com/pone/ and select the 'Submissions Needing Revision' folder to locate your manuscript file.

- A letter that responds to each point raised by the academic editor and reviewer(s). You should upload this letter as a separate file labeled 'Response to Reviewers'.
- A marked-up copy of your manuscript that highlights changes made to the original version. You should upload this as a separate file labeled 'Revised Manuscript with Track Changes'.
- An unmarked version of your revised paper without tracked changes. You should upload this as a separate file labeled 'Manuscript'.

If you would like to make changes to your financial disclosure, please include your updated statement in your cover letter. Guidelines for resubmitting your figure files are available below the reviewer comments at the end of this letter.

If applicable, we recommend that you deposit your laboratory protocols in protocols.io to enhance the reproducibility of your results. Protocols.io assigns your protocol its own identifier (DOI) so that it can be cited independently in the future. For instructions see: https://journals.plos.org/plosone/s/submission-guidelines#loc-laboratory-protocols. Additionally, PLOS ONE offers an option for publishing peer-reviewed Lab Protocol articles, which describe protocols hosted on protocols.io. Read more information on sharing protocols at https://plos.org/protocols?utm_medium=editorial-email&utm_source=authorletters&utm_campaign=protocols.

As the corresponding author, your ORCID iD is verified in the submission system and will appear in the published article. PLOS supports the use of ORCID, and we encourage all coauthors to register for an ORCID iD and use it as well. Please encourage your coauthors to verify their ORCID iD within the submission system before final acceptance, as unverified ORCID iDs will not appear in the published article. *Only*  the individual author can complete the verification step; PLOS staff *cannot*  verify ORCID iDs on behalf of authors.

We look forward to receiving your revised manuscript.

Kind regards,

Jiaci Lin

Academic Editor

PLOS One

Journal Requirements:

If the reviewer comments include a recommendation to cite specific previously published works, please review and evaluate these publications to determine whether they are relevant and should be cited. There is no requirement to cite these works unless the editor has indicated otherwise.

Please review your reference list to ensure that it is complete and correct. If you have cited papers that have been retracted, please include the rationale for doing so in the manuscript text, or remove these references and replace them with relevant current references. Any changes to the reference list should be mentioned in the rebuttal letter that accompanies your revised manuscript. If you need to cite a retracted article, indicate the article’s retracted status in the References list and also include a citation and full reference for the retraction notice.

Additional Editor Comments:

Thank you for submitting your manuscript. I would like to clarify that I am the new editor handling the manuscript following its transfer to me. As only one reviewer evaluated the manuscript in the previous round, I invited an additional reviewer to assess it during the current round. I am pleased to note that the original reviewer has recommended acceptance of the manuscript in its current form. The additional reviewer also provided a positive assessment, together with several comments. Please give careful consideration to these comments and revise the manuscript accordingly.

[Note: HTML markup is below. Please do not edit.]

Reviewers' comments:

Reviewer's Responses to Questions

**Comments to the Author**

Reviewer #1: All comments have been addressed

Reviewer #2: (No Response)

**********

2. Is the manuscript technically sound, and do the data support the conclusions?

Reviewer #1: Yes

Reviewer #2: Yes

**********

3. Has the statistical analysis been performed appropriately and rigorously?

Reviewer #1: Yes

Reviewer #2: (No Response)

**********

4. Have the authors made all data underlying the findings in their manuscript fully available?

The PLOS Data policy

Reviewer #1: Yes

Reviewer #2: Yes

**********

5. Is the manuscript presented in an intelligible fashion and written in standard English?

Reviewer #1: Yes

Reviewer #2: Yes

**********

Reviewer #1: Dear authors,

I believe the authors have responded adequately to my comments.

I have no further comments.

Best regards.

Reviewer #2: This is a well-conducted and timely scoping review that addresses an important public health issue. The manuscript is well-organized, follows the PRISMA-ScR guidelines, and provides a comprehensive overview of changes in mental health outcomes among middle-aged and older adults. However, the authors should address the following points:

Better justify the eligibility criterion “well-being during the COVID-19 pandemic using the same scales for comparability.”

Explicitly acknowledge that no assessment of the methodological quality (risk of bias) of the included studies was performed.

The substantial heterogeneity among the included studies (e.g., countries, follow-up periods, measurement instruments, and study designs) should also be discussed in greater depth, as it limits the comparability of the results.

Throughout the manuscript, use the term “risk factors” with caution. It is appropriate only when discussing longitudinal studies that actually assess true risk. When describing the results of cross-sectional studies, replace it with “factors associated with changes in mental health outcomes,” as these studies do not support causal inferences.

The “Methods” section would also benefit from clarifying whether a review protocol was registered.

Finally, the “Discussion” section should present a more critical interpretation of the results, rather than limiting itself primarily to summarizing the evidence. In particular, the authors should discuss possible explanations for the heterogeneity among the studies.

**********

what does this mean?). If published, this will include your full peer review and any attached files.

If you choose “no”, your identity will remain anonymous but your review may still be made public.

**Do you want your identity to be public for this peer review?**  For information about this choice, including consent withdrawal, please see our Privacy Policy

Reviewer #1: **Yes:** Celso Silva

Reviewer #2: No

**********

[NOTE: If reviewer comments were submitted as an attachment file, they will be attached to this email and accessible via the submission site. Please log into your account, locate the manuscript record, and check for the action link "View Attachments". If this link does not appear, there are no attachment files.]

To ensure your figures meet our technical requirements, please review our figure guidelines: https://journals.plos.org/plosone/s/figures

You may also use PLOS’s free figure tool, NAAS, to help you prepare publication quality figures: https://journals.plos.org/plosone/s/figures#loc-tools-for-figure-preparation.

NAAS will assess whether your figures meet our technical requirements by comparing each figure against our figure specifications.

# Author comment: Author response to Decision Letter 2

**DOI:** 10.1371/journal.pone.0342140.r004

3 Aug 2026

Response to Reviewer Comments

We would like to thank the reviewers for their careful evaluation of our manuscript and their valuable feedback and comments. We have considered all suggestions and have revised the manuscript accordingly. Please find our response to the comments and changes made in the manuscript as follows.

Response to reviewer comments:

Comment 1: Better justify the eligibility criterion “well-being during the COVID-19 pandemic using the same scales for comparability.”

Author response: We have added the following for the justification: “This criterion was applied to ensure that observed changes in mental health outcomes were attributable to changes in mental health status rather than variations in measurement resulting from the use of different scales.” On lines 154- 156

Comment 2: “Explicitly acknowledge that no assessment of the methodological quality (risk of bias) of the included studies was performed”.

Author response: We have added “No review protocol was registered, and no formal assessment of methodological quality (risk of bias) of the included studies was performed [18]” in the methods section, on line 124 to 126

Comment 3: The substantial heterogeneity among the included studies (e.g., countries, follow-up periods, measurement instruments, and study designs) should also be discussed in greater depth, as it limits the comparability of the results.

And finally, the “Discussion” section should present a more critical interpretation of the results, rather than limiting itself primarily to summarizing the evidence. In particular, the authors should discuss possible explanations for the heterogeneity among the studies

Author response: Thank you for the comment. We had mentioned the limitation in terms of periods and measurement in the discussion section: “Another significant limitation of this scoping review is the inclusion of studies employing varied diagnostic tools and effect estimates, which complicates the synthesis of findings and may obscure the overall interpretability and comparability of the results across studies. Studies included in this review exhibit significant variability in both pre-pandemic measurement periods and follow-up periods, affecting cross-study comparability. For instance, a study by Besselaar and colleagues used data from 2011-2019 as a pre-pandemic baseline [28], whereas Amerio and colleagues relied on 2019 data for baseline measures [19], which may affect the comparability across studies.” On lines 440 to 455 of the main manuscript.

We now address the heterogeneity across findings in terms of countries and design by adding the following paragraph (Line 367 to 385 ) to the discussion section: “The substantial heterogeneity in results may be attributable to differences in countries and study designs. Across countries, increases in mental health problems were reported in many settings, including Canada, the United Kingdom, Germany, Japan, Korea, the Faroe Islands, Bangladesh, the Netherlands, Austria, Finland, Chile, and Italy. For example, the majority of Canadian, European, and Asian (excluding China) studies reported worsening mental health outcomes [19–21,23,24,26,28,33,42,44,48,50]. In contrast, studies from the United States and China showed more heterogeneous findings, with some reporting increases [32,51,53,55,58,59], and others reporting decreases or no significant changes [34,36,38,39,45,46]. Outside this region, the study from Brazil reported a decrease in loneliness during the pandemic period [62], and New Zealand showed no significant change [40]. Differences in study design may also explain variation in findings. Most longitudinal studies reported increases in mental health symptoms [20,21,24,26,32,33,42,44,50,55]; however, some studies observed stable or decreased symptoms [34,36,38–40,45,46,62]. In contrast, all included cross-sectional studies showed increases in mental health symptoms during the pandemic; however, interpretation is generally limited because these studies often rely on comparisons with recalled pre-pandemic status [19,23,28,31,43,48,51,53,54]. Overall, the direction and magnitude of changes were not uniform across countries or study designs. The variation suggests that the impact of the pandemic on mental health was influenced not only by the occurrence of COVID-19 itself but also by contextual factors like countries and study design.”

Comment 4: Throughout the manuscript, use the term “risk factors” with caution. It is appropriate only when discussing longitudinal studies that actually assess true risk. When describing the results of cross-sectional studies, replace it with “factors associated with changes in mental health outcomes,” as these studies do not support causal inferences.

Author response: Thank you for bringing this to our attention. The paper summarizes results from a pooled body of both cross-sectional and longitudinal studies, and even for longitudinal studies we cannot guarantee causal inference. Accordingly, we have revised the manuscript to replace “risk factors” with “associated factors” throughout, as our intention in this scoping review is not to establish causal inferences but to identify associated factors.

Comment 5: The “Methods” section would also benefit from clarifying whether a review protocol was registered.

Author response: We have added: “No review protocol was registered, and no formal assessment of methodological quality (risk of bias) of the included studies was performed [18]” in the methods section on line on line 124 to 126

We would once again like to thank the reviewers for their helpful comments and suggestions. These revisions have strengthened our manuscript.

If you require any further revisions or clarifications, please do not hesitate to contact us.

Attachment Submitted filename: Response to Reviewers2.docx

# Editor report: Decision Letter 2

**DOI:** 10.1371/journal.pone.0342140.r005

**Authors:** Jiaci Lin

14 Aug 2026

Changes in mental health outcomes of middle-aged and older adults during the COVID-19 pandemic: A scoping review

PONE-D-26-02556R2

Dear Dr. Wilk,

We’re pleased to inform you that your manuscript has been judged scientifically suitable for publication and will be formally accepted for publication once it meets all outstanding technical requirements.

Within one week, you’ll receive an e-mail detailing the required amendments. When these have been addressed, you’ll receive a formal acceptance letter and your manuscript will be scheduled for publication.

An invoice will be generated when your article is formally accepted. Please note, if your institution has a publishing partnership with PLOS and your article meets the relevant criteria, all or part of your publication costs will be covered. Please make sure your user information is up-to-date by logging into Editorial Manager at Editorial Manager® and clicking the ‘Update My Information' link at the top of the page. For questions related to billing, please contact billing support.

If your institution or institutions have a press office, please notify them about your upcoming paper to help maximize its impact. If they’ll be preparing press materials, please inform our press team as soon as possible -- no later than 48 hours after receiving the formal acceptance. Your manuscript will remain under strict press embargo until 2 pm Eastern Time on the date of publication. For more information, please contact onepress@plos.org.

Kind regards,

Jiaci Lin

Academic Editor

PLOS One

Additional Editor Comments (optional):

Reviewers' comments:

# Editor report: Acceptance letter

**DOI:** 10.1371/journal.pone.0342140.r006

**Authors:** Jiaci Lin

PONE-D-26-02556R2

PLOS One

Dear Dr. Wilk,

I'm pleased to inform you that your manuscript has been deemed suitable for publication in PLOS One. Congratulations! Your manuscript is now being handed over to our production team.

At this stage, our production department will prepare your paper for publication. This includes ensuring the following:

* All references, tables, and figures are properly cited

* All relevant supporting information is included in the manuscript submission,

* There are no issues that prevent the paper from being properly typeset

You will receive further instructions from the production team, including instructions on how to review your proof when it is ready. Please keep in mind that we are working through a large volume of accepted articles, so please give us a few days to review your paper and let you know the next and final steps.

Lastly, if your institution or institutions have a press office, please let them know about your upcoming paper now to help maximize its impact. If they'll be preparing press materials, please inform our press team within the next 48 hours. Your manuscript will remain under strict press embargo until 2 pm Eastern Time on the date of publication. For more information, please contact onepress@plos.org.

You will receive an invoice from PLOS for your publication fee after your manuscript has reached the completed accept phase. If you receive an email requesting payment before acceptance or for any other service, this may be a phishing scheme. Learn how to identify phishing emails and protect your accounts at https://explore.plos.org/phishing.

If we can help with anything else, please email us at customercare@plos.org.

Thank you for submitting your work to PLOS One and supporting open access.

Kind regards,

PLOS ONE Editorial Office Staff

on behalf of

Dr. Jiaci Lin

Academic Editor

PLOS One
